# Microalgal biomass production pathways: evaluation of life cycle environmental impacts

**DOI:** 10.1186/1754-6834-6-88

**Published:** 2013-06-20

**Authors:** George G Zaimes, Vikas Khanna

**Affiliations:** 1 Department of Civil and Environmental Engineering, Swanson School of Engineering, University of Pittsburgh, Pittsburgh, 15261, Pennsylvania

**Keywords:** Microalgae, Biomass, Bioenergy, Biofuel, Life cycle analysis, Chlorella vulgaris, Open raceway ponds, GHG emissions, Water demands

## Abstract

**Background:**

Microalgae are touted as an attractive alternative to traditional forms of biomass for biofuel production, due to high productivity, ability to be cultivated on marginal lands, and potential to utilize carbon dioxide (CO_2_) from industrial flue gas. This work examines the fossil energy return on investment (EROI_fossil_), greenhouse gas (GHG) emissions, and direct Water Demands (WD) of producing dried algal biomass through the cultivation of microalgae in Open Raceway Ponds (ORP) for 21 geographic locations in the contiguous United States (U.S.). For each location, comprehensive life cycle assessment (LCA) is performed for multiple microalgal biomass production pathways, consisting of a combination of cultivation and harvesting options.

**Results:**

Results indicate that the EROI_fossil_ for microalgae biomass vary from 0.38 to 1.08 with life cycle GHG emissions of −46.2 to 48.9 (g CO_2_ eq/MJ-biomass) and direct WDs of 20.8 to 38.8 (Liters/MJ-biomass) over the range of scenarios analyzed. Further anaylsis reveals that the EROI_fossil_ for production pathways is relatively location invariant, and that algae’s life cycle energy balance and GHG impacts are highly dependent on cultivation and harvesting parameters. Contrarily, algae’s direct water demands were found to be highly sensitive to geographic location, and thus may be a constraining factor in sustainable algal-derived biofuel production. Additionally, scenarios with promising EROI_fossil_ and GHG emissions profiles are plagued with high technological uncertainty.

**Conclusions:**

Given the high variability in microalgae’s energy and environmental performance, careful evaluation of the algae-to-fuel supply chain is necessary to ensure the long-term sustainability of emerging algal biofuel systems. Alternative production scenarios and technologies may have the potential to reduce the critical demands of biomass production, and should be considered to make algae a viable and more efficient biofuel alternative.

## Background

Heightened global awareness of climate change and consumption of finite resources has driven research in biomass-based forms of energy production. Current fossil fuel depletion rates and related emissions have prompted development of sustainable energy alternatives that are both carbon neutral and compatible with existing infrastructure. In past years, researchers have examined various biomass feedstocks such as corn, soybean, canola, and lignocellulosic crops for their bioenergy potential. Major drawbacks to these first and second generation biofuels including land use, water footprint, and influence in global food markets have prompted research in alternative forms of biomass [1]. Accordingly, algae-to-energy systems are receiving increased attention from both academic and industrial sectors. Microalgae’s promising characteristics, such as: high productivity [2], ability to be cultivated on marginal lands [3], semi-continuous to continuous harvesting, high lipid content, and potential to utilize carbon dioxide (CO_2_) from industrial flue gas make it an attractive feedstock for biofuel production [4-8]. In addition, microalgae production does not directly displace food crops, as do other leading biomass candidates such as corn or soybean [9]. In 2007, the United States (U.S.) congress passed the Energy Independence and Security Act (EISA), which mandates the production and addition of 36 billion gallons of biofuels to traditional transportation fuels by the year 2022 [10]. Extraction and subsequent upgrading of microalgal biomass feedstock may provide both a liquid fuel that has the potential to be compatible with current transportation fuel infrastructure, and satisfy the EISA mandate.

The prospect of utilizing microalgae for energy production is not a recent phenomenon: between 1978 and 1998 the Unites States Department of Energy’s (DOE) Aquatic Species Program, a research program aimed to develop renewable transportation fuels, extensively examined the production of biodiesel from microalgae [11]. Current demand for transportation fuels, as well as technological advancements and maturation, have motivated researchers to re-examine microalgae’s potential as a fuel source [12], and in recent years have led to a host of microalgae based life cycle assessments (LCA) [13-35]. Prior studies have shown that different algae harvesting options, reactor configurations, culture conditions, and cultivation assumptions yield divergent results concerning algae’s environmental and energy performance [13,15,21-23,26,35,36]. As such, evaluation of the life cycle greenhouse gas (GHG) emissions, fossil energy consumption, and water demands for multiple biomass production pathways within the framework of one study can provide insight into the potential tradeoffs, environmental impacts, and technical feasibility of these pathways. As microalgal derived fuels are inherently dependent on the cultivation of microalgae feedstock, a sustainable pathway for microalgae feedstock production must be identified if algal-based fuels are to become a commercial reality. Furthermore, additional pilot testing and laboratory scale results will be necessary for validating and benchmarking theoretical process modeling [37-39]. Holistic evaluation of emerging algae-to-fuel systems that considers the resource consumption, emissions, and their impact across the entire life cycle is critical to assess the environmental sustainability of emerging algae-based energy systems.

This study examines the critical life cycle energy and environmental drivers in algae cultivation through a comprehensive analysis of a theoretical industrial algae Open Raceway Pond (ORP) facility. Prior studies have indicated that photobioreactors (PBR) have high initial capital and operating costs, which limit their commercial viability [18,40]. For these reasons only ORPs were investigated as a means for mass cultivation of microalgae. This work focuses on a typical process chain for ORPs: cultivation followed by a series of flocculation, dewatering, and additional drying [41]. Algal drying requirements were based on soybean production, where final biomass has a solids concentration of 90% on a weight per weight basis (w/w) [35]. Process energy and material flows were constructed based on first principles of thermodynamics, peer-reviewed literature, heat and material balances, and best available engineering knowledge. Multiple cultivation locations across the United States (U.S.) as well as cultivation and harvesting options are modeled to investigate the extent to which these parameters affect the overall energy balance, GHG emissions, and direct water demand of microalgal biomass production, and identify opportunities for process improvements along the algae supply chain.

This work models the production of microalgal biomass using freshwater algae grown using synthetic fertilizers and CO_2_/flue gas from an industrial power plant. The objective of this work is to compare different technological routes for producing dried algal biomass to be used for as a feedstock for conversion to liquid transportation fuel(s). It is assumed that the biomass must be dried to 90% (w/w) before further downstream processing of biomass-to-fuel is possible and is consistent with current commercially available lipid extraction technologies.

## Methodology and sustainability metrics

### LCA model overview

In this study, a comparative LCA of microalgae cultivation and harvesting options for ORPs was conducted. The scope of the LCA is cradle-to-gate, in which all processes upstream of dried biomass are evaluated. With the exception of polyvinyl chloride (PVC) lining [23], previous LCA studies have shown that algae infrastructure related impacts are negligible as compared to other system processes [16], and were thus excluded from the scope of this study. The functional unit was chosen as one Megajoule (MJ) of dried algal biomass. Cultivation of microalgae was evaluated for 232 National Weather Service (NWS) sites in the continental U.S. [42]. Prior research has suggested that for ORPs, microalgae growth rates rapidly decline when exposed to average temperatures less than 15°C [43]. Of the 232 examined locations, 21 sites were found to have monthly average temperatures within the requisite temperature range required to support the mass cultivation of microalgae. Complete LCA was then conducted for these 21 locations, to examine if variations in regional energy mix as well as climatological and geographical parameters influence algal biomass production. For each cultivation location multiple biomass production pathways were examined, consisting of a combination of two options for CO_2_ procurement (Monoethanolamine (MEA) scrubbing with injection of pure CO_2_ or Direct Injection (DI) of industrial flue gas), two algal dewatering options (centrifugation (CF) or chamber filter press (CFP)), and two algal drying scenarios (natural gas based drying (NGD) or waste heat drying (WHD)).

### Sustainability metrics

The focus of this study is to create an LCA model to evaluate the life cycle energy balance, direct water demands (WD), and net life cycle GHG emissions for the cultivation of microalgae in ORPs. The direct WD was evaluated as the difference between the volume of freshwater required to support algae cultivation and annual regional precipitation. Net life cycle GHG emissions were calculated as the difference between CO_2_ embedded in the microalgae feedstock, as carbon, to the amount of life cycle GHGs emitted throughout the biomass supply chain. As the primary motivation for microalgae production is its potential to displace fossil derived fuels, a fossil energy return on investment metric (EROI_fossil_) was chosen to assess the sustainability of microalgae production. EROI_fossil_, is defined as the ratio of the energy stored in algal biomass (lower heating value x mass of biomass) to the embodied non-renewable fossil energy required to produce algal biomass, and is presented in equation 1.

(1) $$\mathrm{ERO}I_{\mathrm{fossil}}=\frac{\mathrm{Biomass}\;\mathrm{Energy}\;\mathrm{Output}}{\mathrm{Nonrenewable}\;\mathrm{Fossil}\;\mathrm{Energy}\;\mathrm{Input}}$$

Production pathways in which the EROI_fossil_ are greater than 1 are desirable, as more biomass energy is produced than non-renewable fossil energy consumed in biomass production. As the cultivation, dewatering, and harvesting of microalgae is energy intensive and a major bottleneck in the algae-to-fuel production chain [39,44,45], identifying renewable and sustainable pathways for the cultivation of microalgae is critical for the overall advancement of microalgal derived fuels. To reduce the complexity and dimensionality of the data, as well as to allow ease of comparison between different studies, the main paper will provide a detailed analysis and comparison of the net life cycle GHG emissions, direct water demands, and EROI_fossil_ for biomass production pathways for Phoenix, AZ. Detailed tables for EROI_fossil_, direct WD, and GHG emissions for all examined production pathways and locations are provided in the supporting information (see Additional file 1).

### Algal composition and growth rates

An algal growth model was constructed to evaluate microalgal growth rates for ORPs in the continental U.S. Theoretical microalgae photosynthetic yields were constructed based on solar insolation values averaged over a thirty-year period (1961–1990), obtained from the National Solar Radiation Database (NSRD), and efficiency factors determined by pond design and characteristics of the algal culture [46,47]. The fractionated composition of the algae was assumed to be 20% lipids, 25% carbohydrates, 50% proteins, and 5% other organic material and is consistent with previous studies [14,48]. The composition of algae was calculated to be 517 grams (g) C, 81.2 g N, 17.6 g P, and 14.5 g K per kilogram (kg) biomass. The lower heating value (LHV) of the biomass was computed to be 18.66 MJ/kg-biomass.

### Production chain overview and data sources

#### Production chain overview

**Figure 1:**
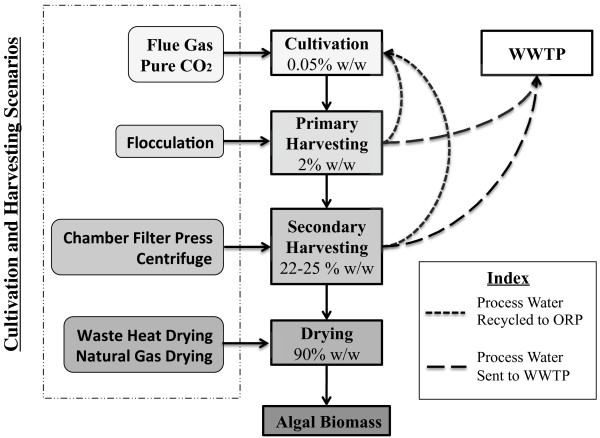
Microalgae biomass production chain and examined production pathways.

Figure 1 shows the microalgal biomass production chain and examined production pathways. Cultivation of the freshwater algae strain, *Chlorella vulgaris*, was modeled in a 1000-hectare (ha) virtual algae production facility, in which 500 ha are allocated for algae cultivation and 500 for infrastructure related demands. This virtual facility was assumed to be colocated with natural gas (NG) fired power plants, and would operate for eight months out of the year, from March to October. *Comprehensive LCA was performed on 21 cultivation locations, spanning seven states: (AL, AZ, CA, FL, GA, LA, & TX).* In each of these locations, biomass production was based on cultivating algae in ORPs. Data concerning the regional electricity mix for the cultivation locations was gathered from the EPA’s “Power Profiler”, based off the 2007 Emissions and Generation Resource Integrated Database (eGRID) [49].

The algal cultivation area is comprised of individual 1-ha ORPs, with a pond depth of 0.3 meters, and operating at an algal concentration of 0.05% (w/w) [17,35]. The algal growth medium circulates at a mixing velocity of 15 cm/second via paddlewheels [41]. A 0.75 mm thick PVC membrane lines the cultivation area [23], with an assumed average lifetime of 5 years [23]. Nutrient and fertilizer requirements were estimated based on algal growth rates and composition of the algal culture. Prior studies have differing assumptions regarding the quantity of nutrients required for algae growth, ranging from approximately one [35] to two [16] times the stoichiometric requirement. In this study a 75% nutrient use-efficiency is assumed, with nitrogen provided by synthetic urea, potassium by potassium chloride, and phosphate by superphosphate. Nutrients and fertilizers are pumped into the ponds with freshwater so that no additional mixing is required. CO_2_ is supplied from a nearby NG fired power plant either by the direct injection of flue gas, or by separating flue gas into pure CO_2_ via MEA scrubbing and delivering pure CO_2_ into the algae ponds [50]. Post cultivation, microalgae are sent to holding tanks, wherein a chemical flocculent, aluminum sulfate, is added to the algal culture to agglomerate the algal biomass so that it can be efficiently separated from the water matrix [51]. The flocculated algae are then sent to either an industrial centrifuge or chamber filter press to concentrate the algae by dewatering. Medium from both flocculation and dewatering stages are recycled back into the ponds to minimize the overall water demands. After dewatering, the microalgae slurry undergoes additional drying. Two scenarios were examined for algal drying. In the first scenario, microalgae are sent to an industrial boiler, in which natural gas is burned to concentrate the algae slurry. The second scenario utilizes waste heat from a colocated power plant as a means of drying the algal biomass. Life cycle data for aluminum sulfate, fertilizers, PVC, and wastewater were taken from the Ecoinvent database [52]. Life cycle data concerning electricity generation and natural gas were taken from the United States Life Cycle Inventory (USLCI) database [53]. Further information regarding algal growth rates, composition, and detailed LCI for all modeled production pathways is available in the supporting information (see Additional file 1).

#### Water demands

The production of biofuels has been shown to be water intensive [54-58]. Quantifying the direct component of the WD can help determine the impacts of biofuel production on regional water resources, and therefore is an important criterion for evaluating optimal locations for algal cultivation. In this study, it is assumed that ORPs are drained and the water is treated at a wastewater treatment facility once every four months to avoid build-up of bacteria and invasive microbes. Additionally, freshwater is required to be pumped into the ponds due to water loss from pond leaking, evaporation, blowdown, photosynthetic requirements, water lost during the harvesting process, algal drying, and water contained within the final biomass that is transported offsite. Water lost due to leaking from the open ponds was evaluated at a rate of 0.27 m^3^/m^2^-year [20]. Evaporative losses were estimated based on the Penman equation [59]. Data for wind speed (m/s), average temperature (°C), and relative humidity (%) averaged over a thirty year period (1961–1990) was obtained from the NSRD [42]. Data concerning average rainfall for the various locations was taken from the National Oceanic and Atmospheric Administration (NOAA) [60]. To avoid excess mineral and salt build-up, and to regulate the pH of the culture medium, a portion of the algal growth medium must be removed from the ponds and replaced with an equivalent amount of freshwater [61]. This process is known as “blowdown”, and it was assumed that onsite evaporation ponds would be used for blowdown disposal. The chemical process of photosynthesis consumes water as a reactant; therefore freshwater that is consumed by photosynthesis in the cultivation ponds must be replaced. During the harvesting stage, process water from both flocculation and dewatering stages are recycled back into the ponds. It was assumed that only 90% [27] of the water recycled from these stages would be returned to the ponds, the remaining 10% must be treated at a wastewater treatment plant (WWTP). Therefore, freshwater must be supplied to the ORPs to offset water that is lost during the harvesting process. Furthermore, freshwater is required to makeup the volume of water that is contained in the final algae biomass that is transported off-site. The direct WD was calculated as the difference between the volume of freshwater required to support algae cultivation and annual precipitation.

## Results and discussion

### EROI_fossil_ and life cycle GHG analysis

Table 1 presents the direct WDs, EROI_fossil_, and net life cycle GHG emissions for all examined biomass production pathways (denoted as A-H) and locations. Table 1 reveals that the net energy balance is negative for a majority of the scenarios analysed. This indicates that more fossil energy is consumed than bioenergy produced during biomass production. Only one out of the eight examined production pathways, (scenario H), was found to yield an EROI_fossil_ greater than 1. Furthermore, scenario H was found to have a barely positive energy balance and is plagued with high technological uncertainty. Additionally, the results reveal that net life cycle GHG emissions are negative for various biomass production pathways, indicating that microalgae sequester more GHGs than are emitted during biomass production via these pathways.

**Table 1:** **EROI**_**fossil**_**, net life cycle GHG emissions, and direct WDs for examined biomass production pathways & locations**

**Scenarios**	A*	B*	C*	D*	E*	F*	G*	H*	**WD**^**1**^	
**Location**	**MEA/CF/NGD**	**MEA/CFP/NGD**	**DI/CF/NGD**	**DI/CFP/NGD**	**MEA/CF/WHD**	**MEA/CFP/WHD**	**DI/CF/WHD**	**DI/CFP/WHD**	**CFP**	**CF**
Mobile, AL	0.40 (44.2)	0.46 (22.5)	0.49 (18.9)	0.59 (−2.8)	0.60 (−0.4)	0.68 (−15.1)	0.86 (−25.7)	1.04 (−40.4)	22.1	22.3
Phoenix, AZ	0.38 (48.9)	0.43 (28.4)	0.47 (23.5)	0.56 (3.0)	0.57 (4.2)	0.64 (−9.2)	0.79 (−21.2)	0.94 (−34.6)	38.6	38.8
San Diego, CA	0.41 (32.0)	0.46 (16.0)	0.51 (6.3)	0.60 (−9.6)	0.63 (−12.6)	0.69 (−21.5)	0.91 (−38.3)	1.06 (−47.2)	32.8	33.0
Daytona Beach, FL	0.38 (43.0)	0.44 (22.7)	0.47 (17.5)	0.57 (−2.7)	0.58 (−1.6)	0.66 (−14.8)	0.81 (−27.1)	0.97 (−40.2)	24.1	24.3
Jacksonville, FL	0.38 (43.1)	0.44 (22.8)	0.47 (17.7)	0.57 (−2.6)	0.58 (−1.5)	0.66 (−14.7)	0.81 (−27.0)	0.97 (−40.1)	22.6	22.9
Key West, FL	0.38 (43.6)	0.44 (23.4)	0.47 (18.2)	0.57 (−2.1)	0.57 (−1.0)	0.65 (−14.2)	0.80 (−26.4)	0.97 (−39.6)	28.4	28.6
Miami, FL	0.38 (42.7)	0.44 (22.5)	0.48 (17.3)	0.57 (−3.0)	0.58 (−1.9)	0.66 (−15.1)	0.81 (−27.3)	0.98 (−40.5)	22.1	22.4
Tallahassee, FL	0.39 (42.4)	0.44 (22.2)	0.48 (17.0)	0.57 (−3.2)	0.58 (−2.2)	0.66 (−15.4)	0.82 (−27.6)	0.98 (−40.8)	20.8	21.1
Tampa, FL	0.38 (43.2)	0.44 (23.0)	0.47 (17.8)	0.57 (−2.5)	0.58 (−1.4)	0.65 (−14.6)	0.81 (−26.8)	0.97 (−40.0)	25.1	25.4
West Palm Beach, FL	0.38 (43.0)	0.44 (22.7)	0.47 (17.6)	0.57 (−2.7)	0.58 (−1.6)	0.66 (−14.8)	0.81 (−27.0)	0.97 (−40.2)	22.9	23.1
Savannah, GA	0.39 (45.0)	0.45 (23.2)	0.49 (19.7)	0.59 (−2.1)	0.60 (0.4)	0.68 (−14.3)	0.86 (−24.9)	1.03 (−39.6)	24.1	24.4
Baton Rouge, LA	0.39 (39.2)	0.45 (20.8)	0.49 (13.6)	0.58 (−4.7)	0.60 (−5.4)	0.67 (−16.8)	0.86 (−31.0)	1.01 (−42.3)	22.6	22.8
Lake Charles, LA	0.39 (39.0)	0.45 (20.6)	0.49 (13.5)	0.58 (−4.9)	0.60 (−5.6)	0.67 (−16.9)	0.86 (−31.1)	1.02 (−42.4)	23.0	23.2
New Orleans, LA	0.40 (38.9)	0.45 (20.5)	0.49 (13.4)	0.58 (−5.0)	0.60 (−5.7)	0.67 (−17.0)	0.86 (−31.2)	1.02 (−42.5)	22.1	22.3
Austin, TX	0.39 (41.5)	0.45 (20.6)	0.49 (16.1)	0.59 (−4.8)	0.59 (−3.1)	0.68 (−16.9)	0.85 (−28.5)	1.03 (−42.3)	29.8	30.0
Brownsville, TX	0.39 (41.8)	0.45 (20.9)	0.49 (16.4)	0.58 (−4.5)	0.59 (−2.8)	0.68 (−16.6)	0.84 (−28.2)	1.02 (−42.0)	30.6	30.8
Corpus Christi, TX	0.39 (42.1)	0.45 (21.3)	0.48 (16.8)	0.58 (−4.1)	0.59 (−2.5)	0.67 (−16.3)	0.84 (−27.8)	1.02 (−41.7)	29.7	29.9
Houston, TX	0.39 (42.0)	0.45 (21.1)	0.49 (16.7)	0.58 (−4.2)	0.59 (−2.6)	0.67 (−16.4)	0.84 (−27.9)	1.02 (−41.8)	25.6	25.8
Lufkin, TX	0.39 (41.5)	0.45 (20.6)	0.49 (16.1)	0.59 (−4.8)	0.59 (−3.1)	0.68 (−16.9)	0.85 (−28.5)	1.03 (−42.3)	26.1	26.3
Port Arthur, TX	0.40 (35.2)	0.46 (16.9)	0.51 (9.7)	0.60 (−8.6)	0.62 (−9.4)	0.70 (−20.6)	0.91 (−34.9)	1.08 (−46.2)	22.8	23.0
San Antonio, TX	0.39 (41.3)	0.45 (20.4)	0.49 (16.0)	0.59 (−4.9)	0.60 (−3.3)	0.68 (−17.1)	0.85 (−28.6)	1.03 (−42.5)	29.9	30.1
Victoria, TX	0.39 (41.9)	0.45 (21.0)	0.49 (16.5)	0.58 (−4.4)	0.59 (−2.7)	0.68 (−16.5)	0.84 (−28.1)	1.02 (−41.9)	27.4	27.7

***MEA*** monoethanolamine, ***DI*** direct injection, ***CF*** centrifuge, ***CFP*** chamber filter press, ***NGD*** natural gas drying, ***WHD*** waste heat drying.

* Values in parentheses represent Net Life Cycle GHG Emissions expressed in unit of (g CO_2_ eq/MJ-Biomass). Values outside of parentheses represent EROI_fossil_. The last two columns represent water demand (WD).

^1^ The results for the WD are presented in units of (liters/MJ-biomass).

A particularly noteworthy observation from Table 1 is that EROI_fossil_ values are relatively location invariant, indicating that changes in regional electricity mix and climatological factors are negligible as compared to other fossil energy intensive processes such as algal biomass drying. Although cultivation locations, such as Arizona, have significantly higher algal growth rates as compared to other examined locations, the energy and GHG impact of producing microalgal feedstock on a per MJ basis for a given production pathway is not significantly different amongst examined locations. However, locations with high aerial biomass productivity may be preferable for algae cultivation as they are capable of generating a greater amount of microalgal biomass feedstock per unit surface area, thus reducing potential land use impacts.

### Direct water demands

Table 1 provides direct WDs for the examined cultivation locations. The results from Table 1 indicate that the WDs of algae cultivation are highly sensitive to geographic location. Moreover, variation in production pathway has negligible effects upon the direct WDs, a trend directly opposite to that observed for EROI_fossil_ and net life cycle GHG emissions. Further analysis indicates that evaporative losses and process water lost during the harvesting stage accounts for the majority of the direct WDs. Additionally, the large variance observed in the direct WDs is primarily due to the large variation in location specific rates of evaporation and precipitation. Additional details on various contributors to water demand for Phoenix, AZ is available in supporting information (see Additional file 1).

### Detailed analysis: Phoenix, Arizona

**Figure 2:**
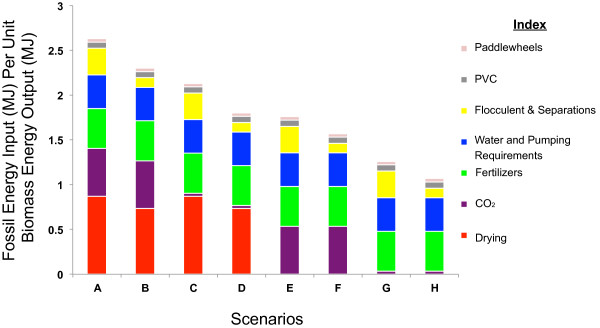
**Life cycle energy analysis for Phoenix, Arizona.** Detailed description for scenarios A-H are provided in Table 1.

**Figure 3:**
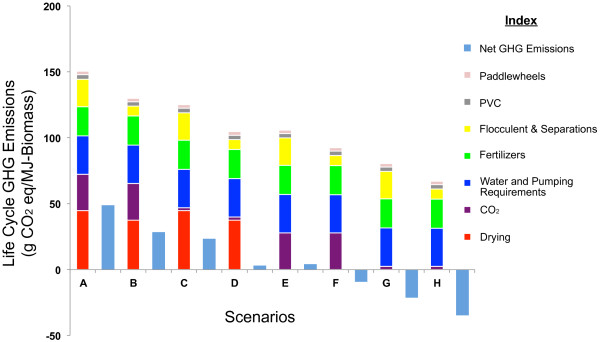
**Life cycle GHG analysis for Phoenix, Arizona.** Detailed description for scenarios A-H are provided in Table 1.

Figure 2 presents the fossil energy inputs normalized per unit of biomass energy output for biomass production pathways for Phoenix, Arizona. For each production pathway, *input* indicates the amount of primary fossil energy consumed for the production of one MJ of biomass energy, *output*. The results highlight that CO_2_ procurement, drying, and fertilizer inputs constitute the largest share of the fossil energy consumption in algal biomass production. In addition these parameters were also found to comprise a high percentage of total life cycle GHG emissions in biomass production as indicated in Figure 3.

### Pure CO_2_ vs. flue gas

The results of this study indicate that the use of MEA-based CO_2_ capture to purify industrial flue gas is energy intensive, primarily due to the high steam requirements for the MEA process. For Phoenix AZ, the primary energy required for the direct injection of industrial flue gas is equivalent to 3.3% of total produced bioenergy. Additionally, life cycle GHG emissions for the direct injection of flue gas were determined to be 2.31 g CO_2_ eq./MJ-biomass. While microalgae’s potential to utilize flue gas as a source of CO_2_ has been extensively cited in the literature [62,63], it remains uncertain if the presence of flue gas will have detrimental effects upon the algae culture [64,65]. There is potential concern that industrial flue gases may contain heavy metals, which may pose serious problems in downstream algal biomass upgrading to transportation fuels. Furthermore, industrial scale operational logistics for the direct injection of flue gas have yet to be evaluated. Therefore, while the utilization of industrial flue gas has the potential to decrease the high energy and environmental cost associated with CO_2_ procurement, the feasibility of direct injection of flue gas on an industrial scale remains questionable, and its effects upon the algal culture are highly uncertain.

### Chamber filter press vs. centrifuge

Chamber filter presses were found to be a more energy efficient method of dewatering, producing a higher concentration biomass (w/w) at a lower energy and environmental cost as compared to centrifugation. For Phoenix, Arizona, switching from centrifugation to chamber filter presses was found to decrease the primary energy consumption of dewatering from approximately 21.4% to 2.4% of total produced bioenergy and decrease related life cycle GHG emissions from 15.0 to 1.65 g CO_2_ eq/MJ-biomass, respectively.

### Natural gas based drying vs. waste heat drying

Natural gas based drying of microalgae was determined to be a critical energy and GHG burden in biomass production. For scenarios utilizing chamber filter presses, the primary energy required for natural gas drying of the microalgae is equivalent to 73% of total produced bioenergy, resulting in life cycle GHG emissions of 37.55 g CO_2_ eq./MJ-biomass. For centrifugation-based pathways the primary energy required for natural gas drying is approximately 87% of total produced bioenergy, with corresponding life cycle GHG emissions of 44.62 g CO_2_ eq./MJ-biomass. Given the high energy and environmental impacts of natural gas based drying, alternate and effective dewatering and drying strategies must be realized. Prior studies have suggested that utilizing waste heat from flue gas streams emanating from colocated power plants could be used to offset algal drying requirements. While the use of this waste heat could considerably decrease algae’s environmental and energy impacts, the technical feasibility and practicality of such a system remains uncertain. Additionally, the quality of the waste heat, and thus its ability to do useful work, are important parameters that may constrain the effectiveness of this approach.

### Sensitivity analysis

**Figure 4:**
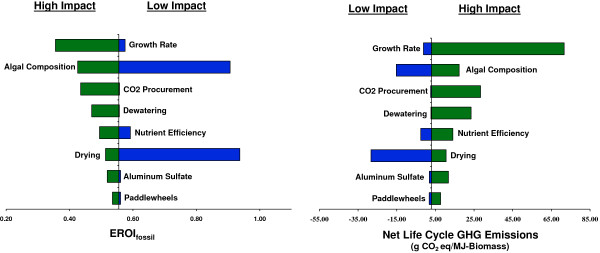
**Sensitivity analysis for Phoenix, Arizona.** Detailed description for sensitivity parameters are provided in Table 2.

A sensitivity analysis was conducted to examine how variations in model parameters influence net life cycle GHG emissions and EROI_fossil_ for algal biomass production. Figure 4 presents tornado plots for EROI_fossil_ values and net life cycle GHG emissions for Phoenix, Arizona. The results in Figure 4 reveal the relative importance and sensitivity of EROI_fossil_ and life cycle GHG emissions to system parameters, and confirm that the cultivation and harvesting of microalgae is highly sensitive to algal composition, CO_2_ procurement, algal growth rates, and drying method. Furthermore, the results suggest that improvements in algae-to-energy production are likely to occur via greater control over algal compositional inputs and advancements in algal drying technologies. Model parameters for sensitivity analysis are provided in Table 2.

**Table 2:** Critical parameters for sensitivity analysis

**Sensitivity analysis**			
**Parameters**	**Low impact**	**Baseline**	**High impact**
**Algal Composition** [% Lipid/Carb/Protein]	50/40/5	20/25/50	5/20/70
**Aluminum Sulfate** [g/m^3^]	80	100	250
**CO**_**2 **_**Procurement** [N/A]	Flue gas (DI) [20% decrease CO_2_ injected]	Flue gas (DI)	Pure CO_2_ (MEA)
**Dewatering** [N/A]	CFP [20% decrease electricity consumption]	CFP	CF
**Drying** [N/A]	WHD	NGD	NGD [20% increase NG consumption]
**Growth Rate** [g/m^2^-day]	35	25	5
**Nutrient Uptake** [%]	100	75	50
**Paddlewheels** [MJ/m^2^-day]	36	65	180

## Conclusions

The results of this study indicate that the life cycle energy and GHG performance of algal biomass production is highly dependent on the production pathway. Analysis reveals that 5 out of the 8 examined production pathways are net GHG negative, while only 1 out of the 8 scenarios have a positive energy balance. Generally, we find that the production of microalgae biomass is energy intensive (reflected in the low EROI), however, the process may be net GHG negative. Furthermore, the life cycle energy balance is found to be relatively location invariant. Contrarily, microalgae’s direct WDs were found to be highly sensitive to geographic location, primarily due to differences in annual precipitation and evaporation. Although regions with high biomass productivity are often touted as optimal locations for microalgae cultivation, they are characteristically found in arid regions with low-freshwater availability. Therefore, quantifying and evaluating the economic and environmental impacts of large scale algae production upon water resources at both the regional and global level is a critical issue that needs to be addressed if algae is to be a commercial source of sustainable bioenergy. Issues of water scarcity, land use change, and land availability may prove to be the constraining factors in commercial bioenergy production. While the direct WD, EROI_fossil_, and net life cycle GHG emissions are important criteria for evaluating biomass feedstocks and biofuels, other sustainability indicators must also be considered to ensure that microalgal derived biofuels do not shift the environmental impacts across their life cycle from one impact category to another.

Thermodynamic constraints dictate that downstream processing and conversion of biomass feedstocks into fuels may only result in further reduction of EROI_fossil_. Therefore, the EROI_fossil_ values for biomass feedstocks may represent an upper bound, or maximum EROI_fossil_ value, for fuels generated via these feedstocks. This study found that the majority of examined microalgal biomass production pathways had a negative energy balance. Subsequently, only one production pathway (H) yielded an EROI_fossil_ value greater than 1, and was found to be only marginally energy positive and plagued with high technological uncertainty, and thus is an indicator that a different approach is necessary. An alternate technological route utilizing auto-flocculation [66], cross flow filtration [67], chamber filter press, as well as natural gas based drying and waste heat drying for producing dried algal biomass was evaluated. Recent studies have suggested that cross flow filtration (CFF) is a low-energy intensive technology that can be used to dewater the algae culture [67], and has many advantages over conventional centrifugation, dissolved air and/or froth flotation [68], and pressure filtration. This technological route may be favorable, as it does not rely on a coagulant for biomass production and uses low-energy dewatering strategies. The EROI_fossil_ and life cycle GHG emissions for this pathway are comparable to scenario (H), detailed results are provided in the supporting information (see Additional file 1).

Improvements in dewatering technologies represent one avenue to decrease microalgae’s high energy burden. For example, in recent years, geosynthetic membranes designed for containment and dewatering of various industrial wastes, have seen commercial application in both wastewater treatment and other industrial processes [69,70]. As these geosynthetic membranes can provide both a low energy and low cost method for dewatering, they may have significant application in algae cultivation. In addition, after use, these geo-synthetic textiles can be recycled and may have a variety of applications in both construction and other industries.

Researchers have suggested coupling wastewater treatment with algae cultivation to reduce the nutrient and freshwater inputs required for algal biomass production and resource inputs necessary for wastewater treatment [16,71,72]. While the use of wastewater for algal biomass cultivation could help minimize algal nutrient requirements, as well as decrease algae’s water footprint [27], studies suggest that waste streams may have relatively low concentrations of both nitrogen and phosphate and thus provide only minor fertilizer offsets [15]. Therefore, the potential of wastewater effluent to offset fertilizer requirements needs further evaluation and validation. Research has suggested that the use of saltwater algae cultures may mitigate algae’s water footprint, however; further research is needed to understand and quantify the potential tradeoffs between sourcing saltwater, land availability, proximity to CO_2_ source, etc. Coupling Geographical Information Systems (GIS) with systems analysis for a realistic evaluation of potential synergies between available land and waste streams (flue gas, wastewater, saltwater) can shed light on the feasibility of large-scale microalgal biomass production [73].

Anaerobic digestion of residual de-oiled biomass (post lipid extraction) has also been suggested as a means of increasing the energy performance of the algae-to-energy system [35,74]. A downfall in this process configuration is that lipid extraction of microalgae feedstock with present-day commercial technology (dry extraction) requires algae to be dried to approximately 90% (w/w), and therefore may be constrained by the energy considerations presented in this study. While the allure of algae based energy is its potential to act as a replacement for traditional transportation fuels, biogas production via anaerobic digestion of the entire algal biomass may have the potential for higher energy yields [75]. One unique advantage of anaerobic digestion is its ability to process wet input streams, and therefore is not limited by algal drying requirements. In addition, recycling of anaerobic digestate may offset a portion of algal nutrient requirements. However, further investigation of the life cycle environmental impacts and benefits of such a system is necessary before a statement can be issued.

This study highlights the importance of systems analysis of emerging algal technologies. Although the need for systems analysis is understood, it receives little attention at early stages of research, often leading to unfounded technological exuberance and optimism. A systems approach with life cycle thinking can test, ground the claims, and assess the environmental sustainability of emerging technologies. Furthermore, systems analysis can aid in identifying technological bottlenecks and sources of process inefficiencies along the supply chain before they become embedded. While industrial symbiosis via the use of wastewater or industrial flue gas and various other synergies have the potential to offset algae’s high cultivation and harvesting costs, with each additional interdependent synergistic technology comes a level of complication that may challenge the performance, reliability, resilience, and viability of the system. The most efficient theoretical system in the end may not provide a practical solution. High-level evaluation of these synergistic opportunities and logistics must be performed in order to assess the commercial viability of algal biofuel systems. As an emerging field, there are many opportunities to enhance the potential of microalgae as an energy source. Alternative production scenarios and technologies may have the potential to reduce the critical demands of microalgal biomass production, and should be considered to make algae a viable and more efficient biofuel alternative.

## Abbreviations

CF: Centrifugation; CFF: Cross flow filtration; CFP: Chamber filter press; CO2: Carbon dioxide; DI: Direct injection of flue gas; DOE: Department of energy; eGRID: Emission and generation resource integrated database; EISA: Energy independence and security act; EROI: Energy return on invenment; GHG: Greenhouse gas; GIS: Geographic information systems; ha: Hectare; LCA: Life cycle assessment; LHV: Lower heating value; MEA: Monoethanolamine; MJ: Megajoule; NG: Natural gas; NGD: Natural gas drying; NOAA: National oceanic and atmospheric administration; NSRD: National solar radiation database; NWS: National weather service; ORP: Open raceway pond; PBR: Photobioreactor; PVC: Polyvinyl chloride; US: United States; USLCI: United States life cycle inventory; WD: Water demands; WHD: Waste heat drying; WWTP: Wastewater treatment plant; w/w: Weight per weight.

## Competing interests

The authors declare that they have no competing interests.

## Authors’ contributions

GGZ constructed the algae life cycle model, analyzed the results, and wrote the manuscript. VK coordinated the study, assisted/guided in the development and analysis of the life cycle model, and reviewed the manuscript. Both authors read and approved the final manuscript.

## Supplementary Material

Additional file 1 **Supporting Info for Microalgae biomass production pathways - Evaluation of life cycle environmental impacts.pdf** Detailed description of the modeling parameters, assumptions, and data used in this study can be found in the supporting document accompanying this article. The supporting information can be accessed free of charge via the journal’s website. Click here for file
