# Supplementary material for: Microalgal biomass production pathways: evaluation of life cycle environmental impacts
Source: Biotechnol Biofuels. 2013 Jun 20;6:88. doi: 10.1186/1754-6834-6-88 (PMC3693881; doi:10.1186/1754-6834-6-88)
Supplement: Additional file 1 — Supporting Info for Microalgae biomass production pathways - Evaluation of life cycle environmental impacts.pdf Detailed description of the modeling parameters, assumptions, and data used in this study can be found in the supporting document accompanying this article. The supporting information can be accessed free of charge via the journal’s website. [file 1754-6834-6-88-S1.pdf]

Supporting Information for:

Microalgal biomass production pathways: Evaluation of life cycle environmental impacts

George G. Zaimes & Vikas Khanna

Table of Contents

1. MODEL OVERVIEW: 3

1.1. RESEARCH OBJECTIVES 3
1.2. REACTOR CONFIGURATION: 3
1.3. METHODOLOGY 3
1.3.1. Fossil Energy Return on Investment ($EROI_{fossil}$) 3
1.3.2. Life Cycle Assessment (LCA) 4
1.4. FUNCTIONAL UNIT AND SYSTEM BOUNDARY 4
1.5. PRODUCTION CHAIN OVERVIEW 5
1.6. CULTIVATION LOCATIONS 6
1.6.1. Electricity Mix 6

2. SOLAR INSOLATION AND CLIMATOLOGICAL DATA 7

2.1. SOLAR INSOLATION 7
2.1.1. Photo-synthetically Active Radiation (PAR) Energy 7
2.2. AUXILIARY CLIMATOLOGICAL PARAMETERS 9
2.3. EVAPORATION 12
2.4. PRECIPITATION 14
2.5. NET WATER ACCUMULATION 14

3. ALGAL BIOMASS 16

3.1. FRACTIONATED BIOMASS COMPOSITION 16
3.2. ALGAL COMPOSITION 16
3.3. ALGAL LOWER HEATING VALUE 18

4. ALGAL GROWTH RATES 19

4.1 EFFICIENCY TERMS 19
4.2. ALGAL GROWTH RATES 21

5. CULTIVATION 22

5.1 CO_2 PROCUREMENT 22
5.1.1 Direct Injection of Flue Gas 22
5.1.2 Monoethanolamine Scrubbing and Injection of Pure CO_2 22
5.2. PADDLEWHEELS 22
5.3. PVC LINER 23
5.4. FRESHWATER SOURCING 23

6. HARVESTING 24

6.1. FLOCCULATION ... 24
6.2. PUMPING REQUIREMENTS ... 24
6.3. CENTRIFUGATION ... 25
6.4. CHAMBER FILTER PRESS ... 26
6.5. ALGAL DRYING ... 26
6.5.1. Natural Gas based Drying ... 26
6.5.2. Waste Heat Drying ... 26

7. LIFE CYCLE INVENTORY ... 27

8. DIRECT WATER DEMANDS ... 32

9. ALLOCATION ... 33

10. SUMMARY TABLES ... 34

11. ADDITIONAL SCENARIOS ... 36

11.1. LOW NITROGEN SCENARIO ... 36
11.2. ALTERNATIVE PRODUCTION SCENARIO ... 39

12. REFERENCE $EROI_{FOSSIL}$ ... 42

13. CITATIONS ... 43

1. MODEL OVERVIEW:

1.1. Research Objectives

The goal of this study is to create an LCA open pond cultivation model to evaluate the direct Water Demands (WD), Fossil Energy Return on Investment ($EROI_{fossil}$), and net life cycle Green House Gas (GHG) emissions for the cultivation of microalgae in open raceway ponds. This study examines multiple cultivation locations as well as cultivation and harvesting options to see if variation in these parameters affects the overall viability of biomass production.

1.2. Reactor Configuration:

Previous studies have shown that the cultivation and harvesting of microalgae is energy intensive and is a major bottleneck in algae-to-biofuel production[1-3]. Reactor configuration is an important parameter that can influence the overall viability of biomass production. While a consensus on the optimal industrial reactor configuration has yet to be established, studies have shown that open raceway pond (ORP) configurations have lower environmental and operating costs than photobioreactors (PBRs)[4, 5]. Past research has also indicated that closed PBR systems have high capital costs, and may not scale efficiently to a commercial level [5, 6]. For these reason's only ORPs were examined in this study.

1.3. Methodology

Life Cycle Analysis (LCA) is utilized as a tool to quantify the environmental and energetic impacts of microalgae biomass production. Energetic Return on investment ($EROI_{fossil}$), which is traditionally used to evaluate fuel types, is implemented as a metric to evaluate the various biomass production pathways.

1.3.1. Fossil Energy Return on Investment ($EROI_{fossil}$)

As the primary motivation for microalgae production (and subsequent downstream processing) is its potential to displace fossil derived fuels, a fossil energy centered metric was chosen to assess the sustainability of microalgae production. In this analysis, the fossil energy return on investment ($EROI_{fossil}$) is defined as the ratio of the amount of energy stored in algal biomass (biomass energy density x mass of feedstock) to the amount of embedded non-renewable fossil energy required to produce algal biomass. $EROI_{fossil}$ (equivalent to the fossil energy ratio[7, 8]) is related to the net energy balance,

which is defined as the difference between the amount of energy stored in biomass to the amount of embedded nonrenewable-fossil energy required to produce biomass. This relationship is highlighted in the following cases:

$EROI_{fossil} > 1$	Energy Balance is Positive
$EROI_{fossil} = 1$	Energy Balance is Zero
$EROI_{fossil} < 1$	Energy Balance is Negative

$EROI_{fossil}$ has distinct significant advantages over contemporary economic analysis. These include: $EROI_{fossil}$ is not affected by market imperfections as is economic analysis, and $EROI_{fossil}$ can be used as a metric to rank the "renewability" of various energy technologies.

1.3.2. Life Cycle Assessment (LCA)

Life Cycle Assessment allows for the quantification of the environmental impacts of a product or service, incorporating the related resource consumption, emissions, and impacts across the various stages of the products life. LCA follows ISO 14040 standards [9].

1.4. Functional Unit and System Boundary

The scope of this LCA is cradle to gate, in which all processes upstream of dried biomass are evaluated. With the exception of PVC lining, previous LCA's have shown that algae infrastructure related impacts are negligible as compared to other system processes[1], and were thus excluded from the scope of this study. The functional unit was chosen as one Mega-Joule (MJ) of dried biomass.

1.5. Production Chain Overview

Figure S1: Microalgal biomass production chain

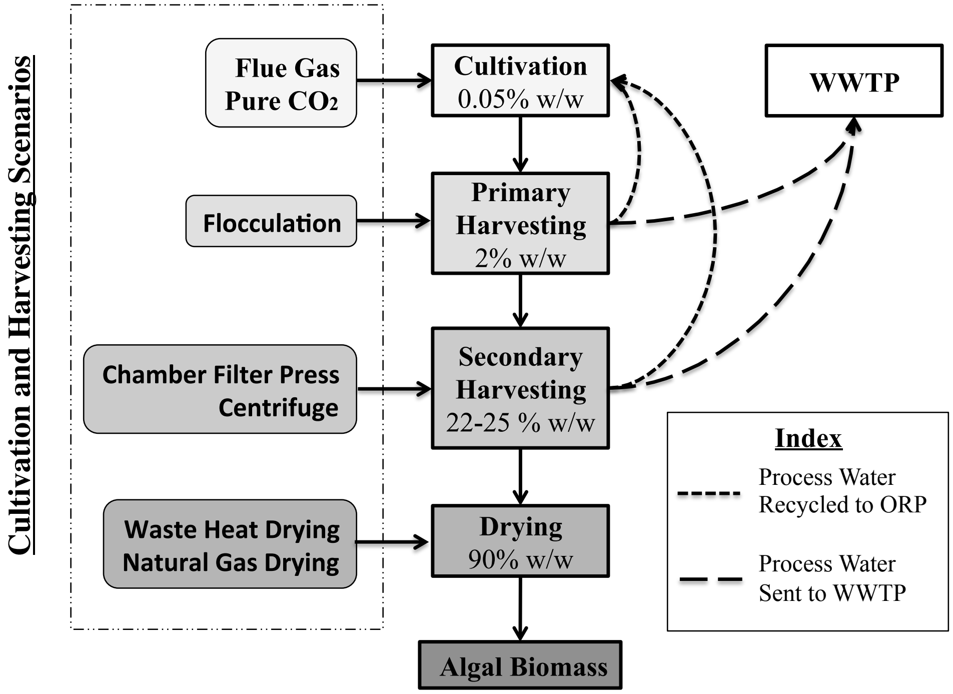

1.6. Cultivation Locations

1.6.1. Electricity Mix

Data concerning the regional electricity mix for the cultivation locations was gathered from the EPA's "Power Profiler", based off the 2007 Emissions and Generation Resource Integrated Database (eGRID), and is presented in Table S1[10]. Life cycle impact factors for regional electricity generation were constructed using the SimaPro software package and eGRID database. Existing USLCI data for electricity generation was modified for each location based on the electricity generation mix (% Coal, Gas, Oil, Nuclear, Hydro, Ren, etc). Impact factors for regional electricity generation were created using the Tool for the Reduction and Assessment of Chemical and other environmental Impacts (TRACI) and cumulative energy demand (CED) methods. Furthermore, electricity distribution losses were accounted for in SimaPro.

Table S1 – Electricity Mix

Resource Mix (%)							
**State**	**City**	**Coal**	**Gas**	**Oil**	**Nuclear**	**Hydro**	**Non-Hydro Renewables**
AL	Mobile	52.2	22.3	0.3	18.1	4.1	2.9
AZ	Phoenix	38.6	35.7	0.1	16.5	6.1	3.1
CA	San Diego	7.3	53	1.4	14.9	12.7	10.1
FL	Daytona Beach	23.7	54.8	4.4	14	0	1.7
FL	Jacksonville	23.7	54.8	4.4	14	0	1.7
FL	Key West	23.7	54.8	4.4	14	0	1.7
FL	Miami	23.7	54.8	4.4	14	0	1.7
FL	Tallahassee	23.7	54.8	4.4	14	0	1.7
FL	Tampa	23.7	54.8	4.4	14	0	1.7
FL	West Palm Beach	23.7	54.8	4.4	14	0	1.7
GA	Savannah	52.2	22.3	0.3	18.1	4.1	2.9
LA	Baton Rouge	22.7	45.1	1.5	26	1.7	1.9
LA	Lake Charles	22.7	45.1	1.5	26	1.7	1.9
LA	New Orleans	22.7	45.1	1.5	26	1.7	1.9
TX	Austin	33	47.8	1.1	12.3	0.2	5.5
TX	Brownsville	33	47.8	1.1	12.3	0.2	5.5
TX	Corpus Christi	33	47.8	1.1	12.3	0.2	5.5
TX	Houston	33	47.8	1.1	12.3	0.2	5.5
TX	Lufkin	33	47.8	1.1	12.3	0.2	5.5
TX	Port Arthur	22.7	45.1	1.5	26	1.7	1.9
TX	San Antonio	33	47.8	1.1	12.3	0.2	5.5
TX	Victoria	33	47.8	1.1	12.3	0.2	5.5

2. SOLAR INSOLATION AND CLIMATOLOGICAL DATA

2.1. Solar Insolation

Data concerning solar insolation for the cultivation locations was gathered from the National Solar Radiation Database (NSRD); this data set contains the average values of solar insolation over a thirty-year period (1961-1990)[11], summarized in

Table S2

Table S2 - Average Solar Insolation (kWh/m^2-day)

State	City	March	April	May	June	July	Aug	Sept	Oct	Average
AL	Mobile	4.4	5.4	5.9	5.9	5.6	5.2	4.7	4.2	5.2
AZ	Phoenix	5.5	7.1	8.0	8.4	7.6	7.1	6.1	4.9	6.8
CA	San Diego	4.9	6.1	6.3	6.5	6.9	6.5	5.4	4.4	5.9
FL	Daytona Beach	5.0	6.2	6.4	6.1	6.0	5.7	4.9	4.2	5.6
FL	Jacksonville	4.7	5.9	6.1	6.0	5.8	5.4	4.6	4.0	5.3
FL	Key West	5.5	6.3	6.3	6.1	6.1	5.8	5.2	4.6	5.7
FL	Miami	5.2	6.0	6.0	5.6	5.8	5.6	4.9	4.4	5.4
FL	Tallahassee	4.7	5.9	6.3	6.1	5.8	5.5	4.9	4.3	5.4
FL	Tampa	5.1	6.2	6.4	6.1	5.8	5.5	4.9	4.4	5.6
FL	West Palm Beach	5.0	5.9	6.0	5.7	5.9	5.6	4.8	4.2	5.4
GA	Savannah	4.7	5.8	6.2	6.3	6.1	5.5	4.7	4.1	5.4
LA	Baton Rouge	4.4	5.4	5.9	6.0	5.7	5.4	4.8	4.3	5.2
LA	Lake Charles	4.5	5.4	6.0	6.3	6.0	5.6	5.0	4.3	5.4
LA	New Orleans	4.5	5.5	6.1	6.1	5.7	5.5	4.9	4.3	5.3
TX	Austin	4.7	5.4	5.9	6.6	6.8	6.3	5.2	4.4	5.7
TX	Brownsville	4.6	5.3	5.8	6.4	6.5	6.0	5.2	4.5	5.5
TX	Corpus Christi	4.4	5.0	5.5	6.1	6.3	5.8	5.0	4.3	5.3
TX	Houston	4.2	5.0	5.6	6.0	5.9	5.6	4.9	4.2	5.2
TX	Lufkin	4.5	5.3	5.9	6.4	6.4	6.0	5.1	4.3	5.5
TX	Port Arthur	4.3	5.2	5.8	6.3	6.1	5.7	5.0	4.3	5.3
TX	San Antonio	4.8	5.5	6.0	6.7	6.9	6.4	5.4	4.5	5.8
TX	Victoria	4.4	5.1	5.7	6.2	6.2	5.8	5.0	4.3	5.3

2.1.1. Photo-synthetically Active Radiation (PAR) Energy

The %PAR (Percent Photo-synthetically Active Radiation) is defined as the ratio of the amount of solar energy that can be utilized in photosynthesis to the full spectrum solar energy, and as such is unit-less. The %PAR was assumed to be 46%, which agrees with previous studies[12], and was assumed to be the same for all examined locations.

Average PAR energy values (kWh/m^2-day) were constructed by multiplying average values of solar insolation (kWh/m^2-day), denoted as I_{avg}, by the %PAR. This is highlighted below, in Equation 1.

Equation 1 $$PARavg = \%PAR * Iavg$$

Substituting in the value for %PAR into Equation 1 yields:

Equation 2 $$PARavg = \left(\frac{46}{100}\right) * Iavg$$

Wherein, PAR_{avg} is the average PAR energy (kWh/m^2-day) and I_{avg} is the value of average solar insolation for the given location and month in units of (kWh/m^2-day). Note: to convert from kWh to MJ multiply by 3.6. The values for average PAR energy (MJ/m^2-day) for various months and locations are presented in Table S3.

Table S3 - Average PAR Energy (MJ/m^2-day)

State	City	March	April	May	June	July	Aug	Sept	Oct	Average
AL	Mobile	7.3	8.9	9.8	9.8	9.3	8.6	7.8	7.0	8.5
AZ	Phoenix	9.1	11.8	13.2	13.9	12.6	11.8	10.1	8.1	11.3
CA	San Diego	8.1	10.1	10.4	10.8	11.4	10.8	8.9	7.3	9.7
FL	Daytona Beach	8.3	10.3	10.6	10.1	9.9	9.4	8.1	7.0	9.2
FL	Jacksonville	7.8	9.8	10.1	9.9	9.6	8.9	7.6	6.6	8.8
FL	Key West	9.1	10.4	10.4	10.1	10.1	9.6	8.6	7.6	9.5
FL	Miami	8.6	9.9	9.9	9.3	9.6	9.3	8.1	7.3	9.0
FL	Tallahassee	7.8	9.8	10.4	10.1	9.6	9.1	8.1	7.1	9.0
FL	Tampa	8.4	10.3	10.6	10.1	9.6	9.1	8.1	7.3	9.2
FL	West Palm Beach	8.3	9.8	9.9	9.4	9.8	9.3	7.9	7.0	8.9
GA	Savannah	7.8	9.6	10.3	10.4	10.1	9.1	7.8	6.8	9.0
LA	Baton Rouge	7.3	8.9	9.8	9.9	9.4	8.9	7.9	7.1	8.7
LA	Lake Charles	7.5	8.9	9.9	10.4	9.9	9.3	8.3	7.1	8.9
LA	New Orleans	7.5	9.1	10.1	10.1	9.4	9.1	8.1	7.1	8.8
TX	Austin	7.8	8.9	9.8	10.9	11.3	10.4	8.6	7.3	9.4
TX	Brownsville	7.6	8.8	9.6	10.6	10.8	9.9	8.6	7.5	9.2
TX	Corpus Christi	7.3	8.3	9.1	10.1	10.4	9.6	8.3	7.1	8.8
TX	Houston	7.0	8.3	9.3	9.9	9.8	9.3	8.1	7.0	8.6
TX	Lufkin	7.5	8.8	9.8	10.6	10.6	9.9	8.4	7.1	9.1
TX	Port Arthur	7.1	8.6	9.6	10.4	10.1	9.4	8.3	7.1	8.8
TX	San Antonio	7.9	9.1	9.9	11.1	11.4	10.6	8.9	7.5	9.6
TX	Victoria	7.3	8.4	9.4	10.3	10.3	9.6	8.3	7.1	8.8

2.2. Auxiliary Climatological Parameters

Data for average wind speed (m/s), average temperature (C), average pressure (kPa), and relative humidity (%) was gathered from the National Solar Radiation Database (NSRD)[11], and are presented in Table S4 to Table S7.

Table S4: Average Pressure

State	City	Pressure (mb)	Pressure (kPa)
**AL**	Mobile	1010	101
**AZ**	Phoenix	974	97.4
**CA**	San Diego	1014	101.4
**FL**	Daytona Beach	1017	101.7
**FL**	Jacksonville	1017	101.7
**FL**	Key West	1016	101.6
**FL**	Miami	1017	101.7
**FL**	Tallahassee	1016	101.6
**FL**	Tampa	1018	101.8
**FL**	West Palm Beach	1017	101.7
**GA**	Savannah	1017	101.7
**LA**	Baton Rouge	1015	101.5
**LA**	Lake Charles	1016	101.6
**LA**	New Orleans	1017	101.7
**TX**	Austin	994	99.4
**TX**	Brownsville	1015	101.5
**TX**	Corpus Christi	1014	101.4
**TX**	Houston	1014	101.4
**TX**	Lufkin	1006	100.6
**TX**	Port Arthur	1017	101.7
**TX**	San Antonio	988	98.8
**TX**	Victoria	1012	101.2

Table S5: Average Wind Speed (m/s)

State	City	Mar	Apr	May	Jun	Jul	Aug	Sep	Oct	Average
**AL**	Mobile	4.7	4.6	3.9	3.4	3	2.9	3.4	3.6	3.7
**AZ**	Phoenix	3.2	3.4	3.4	3.2	3.4	3.2	3	2.8	3.2
**CA**	San Diego	3.5	3.7	3.7	3.6	3.5	3.5	3.4	3.1	3.5
**FL**	Daytona Beach	4.2	4.1	3.8	3.4	3.2	3	3.5	4	3.7
**FL**	Jacksonville	3.9	3.7	3.4	3.2	3	2.8	3.1	3.4	3.3
**FL**	Key West	5.5	5.3	4.8	4.5	4.3	4.1	4.3	5.2	4.8
**FL**	Miami	4.9	4.8	4.4	3.8	3.7	3.7	3.8	4.4	4.2
**FL**	Tallahassee	3.4	3.2	2.9	2.5	2.3	2.3	2.7	2.9	2.8
**FL**	Tampa	4.2	4.1	3.9	3.6	3.3	3.1	3.4	3.8	3.7
**FL**	West Palm Beach	5.1	4.9	4.6	3.9	3.7	3.7	3.9	4.8	4.3
**GA**	Savannah	4.1	3.9	3.4	3.3	3.2	2.9	3.2	3.3	3.4
**LA**	Baton Rouge	4.1	3.9	3.4	2.9	2.6	2.4	2.9	2.9	3.1
**LA**	Lake Charles	4.6	4.5	3.9	3.4	2.9	2.7	3.2	3.4	3.6
**LA**	New Orleans	4.2	4.1	3.6	3	2.6	2.6	3.1	3.3	3.3
**TX**	Austin	4.7	4.5	4.2	3.8	3.5	3.4	3.5	3.5	3.9
**TX**	Brownsville	5.9	6	5.7	5.1	5	4.6	4.2	4.1	5.1
**TX**	Corpus Christi	6.4	6.4	5.7	5	5.1	5	4.9	4.8	5.4
**TX**	Houston	4.4	4.4	3.9	3.6	3.2	3	3.3	3.4	3.7
**TX**	Lufkin	3.6	3.5	3.1	2.6	2.4	2.4	2.6	2.6	2.9
**TX**	Port Arthur	5	5.1	4.4	3.8	3.2	3.1	3.6	3.8	4.0
**TX**	San Antonio	4.5	4.4	4.4	4.3	4.2	3.8	3.8	3.8	4.2
**TX**	Victoria	5.3	5.2	4.9	4.3	4.1	3.8	4	4.1	4.5

Table S6: Average Temperature (°C)

State	City	Mar	Apr	May	Jun	Jul	Aug	Sep	Oct	Average
**AL**	Mobile	15.8	19.9	23.6	26.9	27.9	27.7	25.5	20.2	23.4
**AZ**	Phoenix	16.8	21.1	26.0	31.2	34.2	33.1	29.8	23.6	27.0
**CA**	San Diego	15.3	16.7	17.8	19.3	21.7	22.6	21.9	19.8	19.4
**FL**	Daytona Beach	17.9	20.7	23.7	26.3	27.3	27.2	26.3	23.0	24.1
**FL**	Jacksonville	16.2	19.4	23.0	26.2	27.6	27.3	25.6	21.0	23.3
**FL**	Key West	23.2	25.0	27.0	28.4	29.1	29.1	28.5	26.7	27.1
**FL**	Miami	22.1	24.0	25.9	27.4	28.1	28.2	27.7	25.7	26.1
**FL**	Tallahassee	15.7	19.1	23.1	26.4	27.4	27.4	25.7	20.4	23.2

**FL**	Tampa	19.1	21.8	25.1	27.2	27.8	27.8	27.2	23.8	25.0
**FL**	West Palm Beach	21.1	23.0	25.3	27.0	27.9	28.1	27.6	25.4	25.7
**GA**	Savannah	15.1	18.9	23.1	26.2	27.7	27.2	24.8	19.6	22.8
**LA**	Baton Rouge	16.3	20.5	24.1	26.9	27.9	27.7	25.6	20.3	23.7
**LA**	Lake Charles	15.9	20.2	23.8	26.8	27.9	27.7	25.4	20.6	23.5
**LA**	New Orleans	16.4	20.3	23.8	26.7	27.7	27.5	25.6	20.6	23.6
**TX**	Austin	16.4	20.9	24.2	27.4	29.2	29.3	26.8	21.7	24.5
**TX**	Brownsville	20.4	24.1	26.6	28.3	29.2	29.2	27.7	24.3	26.2
**TX**	Corpus Christi	18.7	22.5	25.5	27.7	28.9	29.0	27.2	23.3	25.4
**TX**	Houston	15.9	20.2	23.6	26.9	28.1	27.9	25.7	20.9	23.7
**TX**	Lufkin	15.3	19.7	23.3	26.6	28.2	28.1	25.2	19.8	23.3
**TX**	Port Arthur	16.3	20.5	24.0	27.1	28.2	28.1	25.9	20.9	23.9
**TX**	San Antonio	16.5	20.7	24.2	27.9	29.4	29.4	26.3	21.2	24.5
**TX**	Victoria	17.4	21.4	24.8	27.6	28.9	28.9	26.4	22.1	24.7

Table S7: Relative Humidity (%)

State	City	Mar	Apr	May	Jun	Jul	Aug	Sep	Oct	Average
**AL**	Mobile	71	70	71	73	76	78	75	71	73
**AZ**	Phoenix	39	28	22	19	32	36	36	37	31
**CA**	San Diego	67	67	71	74	75	74	73	69	71
**FL**	Daytona Beach	71	69	72	77	78	80	79	75	75
**FL**	Jacksonville	71	69	73	77	78	80	81	79	76
**FL**	Key West	73	70	72	74	72	73	75	75	73
**FL**	Miami	69	67	72	76	75	76	78	75	74
**FL**	Tallahassee	72	70	72	76	80	81	78	74	75
**FL**	Tampa	72	69	70	74	77	78	77	74	74
**FL**	West Palm Beach	70	67	71	77	77	76	77	74	74
**GA**	Savannah	67	65	70	74	76	79	78	73	73
**LA**	Baton Rouge	70	71	72	74	77	78	77	73	74
**LA**	Lake Charles	76	76	77	78	80	80	79	76	78
**LA**	New Orleans	73	73	74	76	79	79	78	75	76
**TX**	Austin	64	66	71	69	65	64	68	68	67
**TX**	Brownsville	75	75	77	75	73	74	76	75	75
**TX**	Corpus Christi	74	77	79	78	75	75	76	75	76
**TX**	Houston	73	74	75	75	75	75	76	74	75
**TX**	Lufkin	70	72	75	75	74	73	75	73	73
**TX**	Port Arthur	76	77	79	79	81	80	79	77	79
**TX**	San Antonio	63	66	71	69	65	65	68	67	67
**TX**	Victoria	72	74	76	76	74	74	76	74	75

2.3. Evaporation

Evaporations losses were constructed based on the Penman Equation, show in Equation 3 [13].

Equation 3

$$E_{mass} = \frac{mR_n + \gamma * 6.43 * (1 + 0.563 * U_m) * \delta e}{\lambda_v * (m + \gamma)}$$

Where:
E_{mass} = Evaporation rate (mm day^{-1})
m = Slope of the saturation vapor pressure curve (kPa K^{-1})
R_n = Net Solar radiation (MJ m^{-2} day^{-1})
γ = psychometric constant (kPa K^{-1})
U_m = wind speed (m s^{-1})
δe = vapor pressure deficit (kPa)
λ_v = latent heat of vaporization (MJ kg^{-1})

For average solar insolation (I_{avg}) in units of (watts/m^2), net solar radiation R_n in units of (MJ/m^2-day) is computed using Equation 4 [1].

Equation 4

$$R_n = \left(I_{avg} * \left(\frac{63}{100}\right) - 40\right) * \left(\frac{24}{1000}\right)$$

The latent heat of vaporization (MJ*kg^{-1}) is given by Equation 5 [13]

Equation 5

$$\lambda_v = (2.501 - 0.002361 * Tavg_c)$$

Where: $Tavg_c$ is the average temperature in Celsius

The slopes of the saturation vapor pressure curve in units of (kPa*K^{-1}) are presented in Equation 6.

Equation 6

$$m = \frac{711.5}{Tavg_k{}^2} * \exp\left(21.07 - \frac{5336}{Tavg_k}\right)$$

Where: $Tavg_k$ is the average temperature in Kelvin

The psychometric constant in units of (kPa K^{-1}) is presented in Equation 7 [13].

Equation 7 $$\gamma = \frac{.0016286 * Pavg_{kPa}}{\lambda_v}$$

Where: $Pavg_{kPa}$ is the mean pressure for the given location in units of kPa. The vapor pressure deficit in units of (kPa), denoted δe, is given by Equation 8.

Equation 8 $$\delta e = (e_s - e_a) = (1 - relative\ humidity) * e_s$$

Where: e_s is the saturated vapor pressure of air (kPa), e_a is the vapor pressure of free flowing air (kPa), relative humidity (%).

The saturation vapor pressure of air in units of (kPa), denoted e_s, is approximated in Equation 9 [14].

Equation 9 $$e_s = \frac{1}{7.5} * \exp\left(21.07 - \frac{5336}{Tavg_k}\right)$$

Evaporative losses (mm/day) are presented in Table S8.

.

Table S8: Evaporative Losses (mm/day)

State	City	Mar	Apr	May	Jun	Jul	Aug	Sep	Oct	Average
AL	Mobile	3.5	4.5	4.9	4.9	4.4	4.0	3.8	3.4	4.2
AZ	Phoenix	5.4	7.7	9.5	10.6	9.7	8.7	7.4	5.6	8.1
CA	San Diego	3.6	4.5	4.5	4.6	5.0	4.9	4.1	3.5	4.3
FL	Daytona Beach	3.9	5.0	5.2	4.7	4.6	4.2	3.8	3.5	4.4
FL	Jacksonville	3.5	4.6	4.7	4.6	4.4	3.9	3.3	2.8	4.0
FL	Key West	5.0	5.9	5.7	5.5	5.6	5.2	4.7	4.4	5.2
FL	Miami	4.8	5.6	5.3	4.6	4.9	4.7	4.0	3.9	4.7
FL	Tallahassee	3.3	4.3	4.7	4.5	4.1	3.8	3.6	3.1	3.9
FL	Tampa	4.0	5.1	5.5	5.0	4.6	4.2	3.9	3.7	4.5
FL	West Palm Beach	4.6	5.5	5.4	4.6	4.8	4.7	4.0	4.0	4.7
GA	Savannah	3.7	4.8	5.0	5.0	4.8	4.1	3.5	3.1	4.2
LA	Baton Rouge	3.4	4.3	4.7	4.7	4.3	4.0	3.6	3.2	4.0
LA	Lake Charles	3.2	4.1	4.6	4.8	4.4	4.1	3.7	3.2	4.0
LA	New Orleans	3.4	4.3	4.8	4.7	4.2	4.0	3.7	3.2	4.0
TX	Austin	4.2	4.9	5.1	5.8	6.3	6.0	4.7	3.8	5.1
TX	Brownsville	4.1	4.9	5.2	5.8	6.1	5.5	4.5	3.8	5.0
TX	Corpus Christi	4.0	4.5	4.7	5.2	5.8	5.4	4.5	3.8	4.7
TX	Houston	3.2	4.0	4.4	4.9	4.8	4.5	3.9	3.2	4.1
TX	Lufkin	3.3	4.0	4.4	4.8	4.9	4.7	3.8	3.0	4.1

**TX**	Port Arthur	3.2	4.1	4.5	4.9	4.5	4.3	3.8	3.2	4.1
**TX**	San Antonio	4.2	4.9	5.2	6.1	6.7	6.2	4.9	4.0	5.3
**TX**	Victoria	3.7	4.4	4.8	5.2	5.4	5.0	4.2	3.6	4.6

2.4. Precipitation

Data concerning average rainfall for the various locations was taken from the National Oceanic and Atmospheric Administration (NOAA)[15], the average rainfall (mm/day) for the cultivation locations is presented in Table S9.

Table S9: Average Rainfall (mm/day)

State	City	Mar	Apr	May	Jun	Jul	Aug	Sep	Oct	Average
**AL**	Mobile	5.9	4.1	5.0	4.1	5.4	5.1	4.9	2.7	4.6
**AZ**	Phoenix	0.9	0.2	0.1	0.1	0.8	0.8	0.6	0.6	0.5
**CA**	San Diego	1.9	0.6	0.2	0.1	0.0	0.1	0.2	0.4	0.4
**FL**	Daytona Beach	3.1	2.1	2.7	4.7	4.2	5.0	5.4	3.7	3.9
**FL**	Jacksonville	3.2	2.6	2.9	4.4	4.9	5.6	6.5	3.2	4.2
**FL**	Key West	1.5	1.7	2.9	3.7	2.7	4.4	4.5	3.6	3.1
**FL**	Miami	2.1	2.8	4.5	7.0	4.7	7.1	6.9	5.1	5.0
**FL**	Tallahassee	5.3	2.9	4.1	5.7	6.6	5.8	4.1	2.7	4.6
**FL**	Tampa	2.3	1.5	2.3	4.5	5.3	6.2	5.4	1.9	3.7
**FL**	West Palm Beach	3.0	2.9	4.4	6.2	4.9	5.4	6.6	4.5	4.8
**GA**	Savannah	3.0	2.7	3.0	4.5	4.9	5.9	4.2	2.6	3.8
**LA**	Baton Rouge	4.2	4.6	4.4	4.4	4.9	4.8	4.0	3.1	4.3
**LA**	Lake Charles	2.9	3.0	5.0	5.0	4.2	4.0	4.9	3.2	4.0
**LA**	New Orleans	4.3	4.1	3.8	5.6	5.1	5.0	4.5	2.5	4.4
**TX**	Austin	1.8	2.1	4.1	3.1	1.6	1.9	2.4	3.3	2.5
**TX**	Brownsville	0.8	1.6	2.0	2.4	1.5	2.4	4.4	3.1	2.3
**TX**	Corpus Christi	1.4	1.7	2.9	2.9	1.6	2.9	4.1	3.2	2.6
**TX**	Houston	2.8	2.9	4.2	4.4	2.6	3.1	3.5	3.7	3.4
**TX**	Lufkin	3.0	3.2	4.0	3.1	2.6	2.5	2.8	2.7	3.0
**TX**	Port Arthur	3.1	3.1	4.8	5.4	4.3	4.0	5.0	3.8	4.2
**TX**	San Antonio	1.5	2.1	3.9	3.5	1.7	2.1	2.5	3.2	2.6
**TX**	Victoria	1.8	2.4	4.2	4.1	2.4	2.5	4.1	3.5	3.1

2.5. Net Water Accumulation

The net water accumulation (mm/day) was calculated as the difference between the average rainfall (mm/day) and evaporative losses (mm/day), as shown in Equation 10.

Equation 10 $$NetWaterAccum = AvgRainfall - EvapLoss$$

Negative values signify a net negative water accumulation, indicating that additional water must be pumped into the ponds. The values for net water accumulation for the various locations are shown in Table S10.

Table S10: Net Water Accumulation (mm/day)

State	City	Mar	Apr	May	Jun	Jul	Aug	Sep	Oct	Average
AL	Mobile	2.4	-0.2	0.1	-0.6	0.9	1.1	1.3	-0.7	0.5
AZ	Phoenix	-4.5	-7.5	-9.4	-10.5	-8.9	-8.0	-6.8	-5.0	-7.6
CA	San Diego	-1.8	-3.9	-4.4	-4.5	-5.0	-4.8	-4.0	-3.1	-3.9
FL	Daytona Beach	-0.8	-2.9	-2.5	0.1	-0.4	0.8	1.8	0.2	-0.4
FL	Jacksonville	-0.3	-1.9	-1.8	0.0	0.5	1.7	3.4	0.3	0.2
FL	Key West	-3.5	-4.2	-2.9	-1.6	-2.9	-0.8	-0.1	-0.8	-2.1
FL	Miami	-2.7	-2.8	-0.8	2.6	-0.1	2.4	3.1	1.1	0.4
FL	Tallahassee	2.0	-1.3	-0.7	1.4	2.5	1.9	0.7	-0.4	0.8
FL	Tampa	-1.7	-3.6	-3.1	-0.4	0.7	2.0	1.6	-1.8	-0.8
FL	West Palm Beach	-1.5	-2.5	-1.0	1.8	0.1	0.8	2.8	0.5	0.1
GA	Savannah	-0.7	-2.0	-2.0	-0.4	0.1	1.8	0.8	-0.5	-0.4
LA	Baton Rouge	0.7	0.4	-0.3	-0.2	0.6	0.8	0.5	0.0	0.3
LA	Lake Charles	-0.3	-1.0	0.4	0.3	-0.2	-0.1	1.3	0.1	0.1
LA	New Orleans	0.9	0.0	-1.0	1.1	0.9	1.0	1.0	-0.7	0.4
TX	Austin	-2.4	-2.7	-0.9	-2.6	-4.7	-4.1	-2.3	-0.5	-2.5
TX	Brownsville	-3.3	-3.2	-3.2	-3.3	-4.6	-3.0	0.0	-0.7	-2.7
TX	Corpus Christi	-2.6	-2.7	-1.9	-2.2	-4.1	-2.5	-0.3	-0.6	-2.1
TX	Houston	-0.4	-0.9	-0.2	-0.4	-2.2	-1.3	-0.2	0.5	-0.6
TX	Lufkin	-0.2	-0.7	-0.4	-1.6	-2.3	-2.2	-0.9	-0.3	-1.1
TX	Port Arthur	-0.2	-0.8	0.3	0.7	-0.2	-0.3	1.3	0.6	0.2
TX	San Antonio	-2.7	-2.7	-1.3	-2.5	-5.0	-4.0	-2.4	-0.8	-2.7
TX	Victoria	-1.9	-1.9	-0.6	-1.0	-3.0	-2.5	0.0	-0.1	-1.4

3. ALGAL BIOMASS

3.1. Fractionated Biomass Composition

The composition and net calorific value of the biomass fractions were taken from Lardon et al. 2009, and are presented in Table S11[16].

Table S11: Biomass Content

	Fraction	Composition	Molar Mass ($g*mole^{-1}$)	Net Calorific Value (MJ/g- biomass)
1	Protein	$C_{4.43}H_7O_{1.44}N_{1.16}$	100.1	$15.5*10^{-3}$
2	Carbohydrate	$C_6H_{12}O_6$	180	$13*10^{-3}$
3	Lipid	$C_{40}H_{74}O_5$	634	$38.3*10^{-3}$

3.2. Algal Composition

The algae composition was assumed to be 50% proteins, 25% carbohydrates, 20% lipids, and 5% other organic material, which correlates with previous studies [17, 18]. It was assumed that the values of P, K, Mg, and S vary linearly with the protein content; a proportionality constant was constructed based on a reference composition of *Chlorella vulgaris*, obtained from Lardon et al. 2009[16]. The algae compositional parameters are provided in Table S12, additionally information on reported algal composition, lipid content, and productivies are provided in Table S13 and Table S14.

Table S12: Algae Composition

	Parameters	Reference Composition (g/kg-biomass)	Composition assumed in this study (g/kg-biomass)
1	Protein	282	500
2	Carbohydrate	495	250
3	Lipid	175	200
4	C	480	517
5	N	46	81.2
6	P	9.9	17.6
7	K	8.2	14.5
8	Mg	3.8	6.7
9	S	2.2	3.9

Table S13 – Fractionated Biomass composition for different algal biomass strains (% dry matter)

Parameters	Protein	Carbohydrates	Lipids	Nucleic acid
Aphanizomenon flos-aquae	62	23	3	-
Anabaena cylindrica	43-56	25-30	4-7	-
Arthrospira maxima	60–71	13–16	6–7	-
Chlamydomonas rheinhardii	48	17	21	-
Chlorella pyrenoidosa	57	26	2	-
Chlorella vulgaris	51-58	12-17	14-22	4-5
Dunaliella bioculata	49	4	8	-
Dunaliella salina	57	32	6	-
Euglena gracilis	39-61	14-18	14-20	-
Porphyridium cruentum	28-39	40-57	9-14	-
Prymnesium parvum	28-45	25-33	22-38	1-2
Scenedesmus dimorphus	8-18	21-52	16-40	-
Scenedesmus obliquus	50-56	10-17	12-14	3-6
Scenedesmus quadricauda	47	-	1.9	-
Spirogyra sp.	6-20	33-64	11-21	-
Spirulina maxima	60-71	13-16	6-7	3-4.5
Spirulina platensis	46-63	8-14	4-9	2-5
Synechoccus sp.	63	15	11	5
Tetraselmis maculata	52	15	3	-

Adopted from: ref[18, 19]

Table S14 – Lipid content and productivity for select microalgae strains

Microalgae species	Marine or Freshwater	Lipid content (% dry weight)	Lipid productivity (mg/L/day)	Volumetric productivity (g/L/day)
Ankistrodesmus sp.	Freshwater	24.0-31.0	-	-
Botryococcus braunii	Freshwater	25.0-75.0	-	0.02
Chaetoceros muelleri	Marine	33.6	21.8	0.07
Chaetoceros calcitrans	Marine	14.6-16.4/39.8	17.6	0.04
Chlorella emersonii	Freshwater	25.0-63.0	10.3-50.0	0.036-0.041
Chlorella protothecoides	Freshwater	14.6-57.8	1214	2.00-7.70
Chlorella sorokiniana	Freshwater	19.0-22.0	44.7	0.23-1.47
Chlorella vulgaris	Freshwater	5.0-58.0	11.2-40.0	0.02-0.20
Chlorella sp.	Freshwater	10.0-48.0	42.1	0.02-2.5
Chlorella pyrenoidosa	Freshwater	2	-	2.90-3.64
Chlorella	Freshwater	18.0-57.0	18.7	-
Chlorococcum sp.	Freshwater	19.3	53.7	0.28
Crypthecodinium cohnii	Marine	20.0-51.1	-	10
Dunaliella salina	Marine	6.0-25.0	116	0.22-0.34
Dunaliella primolecta	Marine	23.1	-	0.09
Dunaliella tertiolecta	Marine	16.7-71.0	-	0.12
Dunaliella sp.	Marine	17.5-67.0	33.5	-
Ellipsoidion sp.	Freshwater	27.4	47.3	0.17
Euglena gracilis	Freshwater	14.0-20.0	-	7.7
Haematococcus pluvialis	Freshwater	25	-	0.05-0.06
Isochrysis galbana	Marine	7.0-40.0	-	0.32-1.60
Isochrysis sp.	Marine	7.1-33	37.8	0.08-0.17

Monodus subterraneus	Freshwater	16	30.4	0.19
Monallanthus salina	Marine	20.0-22.0	-	0.08
Nannochloris sp.	Freshwater	20.0-56.0	60.9-76.5	0.17-0.51
Nannochloropsis oculata.	Freshwater	22.7-29.7	84.0-142.0	0.37-0.48
Nannochloropsis sp.	Freshwater	12.0-53.0	37.6-90.0	0.17-1.43
Neochloris oleoabundans	Freshwater	29.0-65.0	90.0-134.0	-
Nitzschia sp.	Freshwater	16.0-47.0	-	-
Oocystis pusilla	Freshwater	10.5	-	-
Pavlova salina	Marine	30.9	49.4	0.16
Pavlova lutheri	Marine	35.5	40.2	0.14
Phaeodactylum tricornutum	Marine	18.0-57.0	44.8	0.003-1.9
Porphyridium cruentum	Marine	9.0-18.8/60.7	34.8	0.36-1.50
Scenedesmus obliquus	Freshwater	11.0-55.0	-	0.004-0.74
Scenedesmus quadricauda	Freshwater	1.9-18.4	35.1	0.19
Scenedesmus sp.	Freshwater	19.6-21.1	40.8-53.9	0.03-0.26
Skeletonema sp.	Marine	13.3-31.8	27.3	0.09
Skeletonema costatum	Marine	13.5-51.3	17.4	0.08
Spirulina platensis	Freshwater	4.0-16.6	-	0.06-4.3
Spirulina maxima	Freshwater	4.0-9.0	-	0.21-0.25
Thalassiosira pseudonana	Marine	20.6	17.4	0.08
Tetraselmis suecica	Marine	8.5-23.0	27.0-36.4	0.12-0.32
Tetraselmis sp.	Marine	12.6-14.7	43.4	0.3

Adopted from: ref[20]

3.3. Algal Lower Heating Value

The energetic content of the algae in units of MJ/kg-biomass, denoted as β, was calculated as the sum of the individual biomass fractions (g/kg biomass) multiplied by their respective energetic content (MJ/g). The biomass Lower Heating Value (LHV) was computed to be 18.66 MJ/kg-biomass.

4. Algal Growth Rates

Algae growth rates were calculated based on the amount of PAR energy a region receives as well as efficiency terms determined by both pond design and characteristics of the algae culture.

4.1 Efficiency Terms

Photosynthetic Efficiency: accounts for the efficiency of converting solar energy into chemical energy by the process of photosynthesis. The equation governing photosynthesis is presented in Equation 11. The photosynthetic efficiency is determined by the quantum requirement, average photon energy, and carbohydrate energy content, and is presented in Equation 12. Values for quantum requirement, average photon energy, and carbohydrate energy content were taken from Weyer et al. 2010[12].

Equation 11

$$CO_2 + H_2O + 8\ photons \rightarrow CH_2O + O_2$$

a. *Quantum Requirement* (Mole Photons/Mole CH_2O): represents the number of photons needed to produce a photosynthetic reaction, this value was assumed to be 8 moles of photons per mole of CH_2O. [12]

b. *Average Photon Energy* (MJ/Mole Photons): corresponds to the average photonic energy of solar radiation, this value was assumed to be $225.3*10^{-3}$ MJ per mole of photons. [12]

c. *Carbohydrate Energy Content* (MJ/Mole CH_2O): represents the energetic content of CH_2O formed in photosynthesis, this value was taken to be $482.5*10^{-3}$ MJ per mole of CH_2O. [12]

Equation 12

$$Photosynthetic\ Efficiency\ (\%) = \left(\frac{Carbohydrate\ Enery\ Content}{Average\ Photon\ Energy\ x\ Quantum\ Requirement}\right) x100$$

Values for photosynthetic efficiency, quantum requirement, average photon energy, and carbohydrate energy content are presented in Table S15.

Table S15: Photosynthetic Efficiency Terms

Term	Value
**Photosynthetic Efficiency (%)**	26.8
**Quantum Requirement (Moles Photons/Moles CH_2O):**	8
**Average Photon Energy (MJ/Mole Photons):**	$225.3*10^{-3}$
**Carbohydrate Energy Content (MJ/Mole CH_2O):**	$482.5*10^{-3}$

Losses due to Reflection: accounts for solar radiation reflected off of the pond surface. For the months of March through October, and for regions between 20-30 degrees latitude, the percent of solar radiation reflected off of the pond surface ranges between 6-8%[21]. This corresponds to an average efficiency value of 93%.

Losses due to sub-optimal environmental conditions: accounts for losses in photon absorption due to temperature and environmental conditions, this value was taken to be 95%. [12]

Photon Utilization Efficiency: accounts for losses in photon absorption in the algal culture due to high or low light levels. For low light levels, photon utilization typically varies between 50-90%, for high light levels 10-30% [22]. For the open pond system, the photon utilization efficiency was taken to be 25%.

Biomass accumulation efficiency: Energy that is available to the algae culture will be used either in cellular respiration or will be stored as biomass. The biomass accumulation efficiency is the ratio of the amount of energy stored in the biomass to the total energy available to the algal culture, and thus shows the efficiency at which algae convert available energy into biomass, this value was taken to be 72%[23].

The values for the efficiency terms are shown in Table S16.

Table S16: Efficiency Factors

	**Efficiency Factors**	**Value (%)**
**1**	Photosynthetic efficiency:	26.8
**2**	Losses due to reflection:	93
**3**	Losses due to sub-optimal environmental conditions:	95
**4**	Photon utilization efficiency:	25
**5**	Biomass accumulation efficiency:	72

Let α denote the product of the five efficiency terms, highlighted in Equation 13.

Equation 13 $$\alpha = \prod_{i=1}^{i=5} EfficienyFactors_i$$

Computing this value, we find that

Equation 14 $$\alpha = 4.262 * 10^{-2}$$

Let us denote another quantity, δ (g/MJ), equal to the ratio of α (unit-less) to the lower heating value β (MJ/g), given by Equation 15.

Equation 15 $$\delta = \frac{\alpha}{\beta}$$

4.2. Algal Growth Rates

The growth rates in units of (g/m^2-day) of the algae culture were calculated as the product of the average PAR energy (MJ/m^2-day) and δ (g/MJ), expressed in Equation 16.

Equation 16 $$Growth\ Rates = PARavg * \delta$$

Monthly average micro-algal growth rates for all examined locations are presented in Table S17

Table S17: Algae Growth Rates (g/m^2-day)

State	City	Mar	Apr	May	Jun	Jul	Aug	Sep	Oct	Average
**AL**	Mobile	16.6	20.4	22.3	22.3	21.2	19.6	17.8	15.9	19.5
**AZ**	Phoenix	20.8	26.8	30.2	31.7	28.7	26.8	23.0	18.5	25.8
**CA**	San Diego	18.5	23.0	23.8	24.6	26.1	24.6	20.4	16.6	22.2
**FL**	Daytona Beach	18.9	23.4	24.2	23.0	22.7	21.5	18.5	15.9	21.0
**FL**	Jacksonville	17.8	22.3	23.0	22.7	21.9	20.4	17.4	15.1	20.1
**FL**	Key West	20.8	23.8	23.8	23.0	23.0	21.9	19.6	17.4	21.7
**FL**	Miami	19.6	22.7	22.7	21.2	21.9	21.2	18.5	16.6	20.5
**FL**	Tallahassee	17.8	22.3	23.8	23.0	21.9	20.8	18.5	16.2	20.5
**FL**	Tampa	19.3	23.4	24.2	23.0	21.9	20.8	18.5	16.6	21.0
**FL**	West Palm Beach	18.9	22.3	22.7	21.5	22.3	21.2	18.1	15.9	20.4
**GA**	Savannah	17.8	21.9	23.4	23.8	23.0	20.8	17.8	15.5	20.5
**LA**	Baton Rouge	16.6	20.4	22.3	22.7	21.5	20.4	18.1	16.2	19.8
**LA**	Lake Charles	17.0	20.4	22.7	23.8	22.7	21.2	18.9	16.2	20.4
**LA**	New Orleans	17.0	20.8	23.0	23.0	21.5	20.8	18.5	16.2	20.1
**TX**	Austin	17.8	20.4	22.3	24.9	25.7	23.8	19.6	16.6	21.4
**TX**	Brownsville	17.4	20.0	21.9	24.2	24.6	22.7	19.6	17.0	20.9
**TX**	Corpus Christi	16.6	18.9	20.8	23.0	23.8	21.9	18.9	16.2	20.0
**TX**	Houston	15.9	18.9	21.2	22.7	22.3	21.2	18.5	15.9	19.6
**TX**	Lufkin	17.0	20.0	22.3	24.2	24.2	22.7	19.3	16.2	20.7
**TX**	Port Arthur	16.2	19.6	21.9	23.8	23.0	21.5	18.9	16.2	20.2
**TX**	San Antonio	18.1	20.8	22.7	25.3	26.1	24.2	20.4	17.0	21.8
**TX**	Victoria	16.6	19.3	21.5	23.4	23.4	21.9	18.9	16.2	20.2

5. Cultivation

5.1 CO_2 Procurement

CO_2 from a nearby natural gas fired power plant is supplied to the ponds in two ways:

5.1.1 Direct Injection of Flue Gas

(i) Flue gas is transported via lower pressure blowers and delivered to the algae ponds, evaluated at 22.2×10^{-3} kilowatt hours (kWh) per kg CO_2 [24]. While microalgae's potential to utilize flue gas as a source of CO_2 has been extensively cited in the literature [25, 26], it remains uncertain if the presence of flue gas will have detrimental effects upon the algae culture [27, 28]. More so, there is potential concern that industrial flue gases may contain heavy metals, which may decrease the quality of algal derived fuels. In this study, it is assumed that the Direct Injection (DI) of flue gas has no negative impacts upon the algae culture. While the utilization of industrial flue gas has the potential to decrease the high energetic cost associated with CO_2, the feasibility of direct injection of flue gas on an industrial scale remains questionable.

5.1.2 Monoethanolamine Scrubbing and Injection of Pure CO_2

(ii) Flue gas is separated into pure CO_2 via Monoethanolamine (MEA) scrubbing; this pure CO_2 is then delivered to the ponds via low-pressure blowers. Kadam et al. 2002 estimated that 1 kg of CO_2 from MEA extraction would require approximately 2.01 kg of steam and 32.65×10^{-3} kWh of electricity [24]. The energy required to transform water to steam was based on the enthalpy of steam, evaluated at 2.6 MJ/kg-steam. It was assumed that natural gas would be burned to generate steam; the energetic content of natural gas was taken to be 39 MJ/m^3-natural gas and a boiler efficiency of 80% was assumed[29]. While MEA scrubbing is the more energy intensive of the two options, it insures that the algal culture does not experience the possible negative effects as associated with the direct injection of flue gas.

It this study, it was assumed that the microalgae culture captures only 70% of the injected CO_2 [4].

5.2. Paddlewheels

During cultivation, the algal growth medium is circulated by paddlewheels, consistent with current reactor configurations [1, 5, 30]. While other medium circulation configurations have been proposed, paddlewheels are a proven technology, and appear to be the most effective method of circulating the algal growth medium. For a mixing velocity of 15 cm/second and a pond depth of 0.3 m, the energetic cost of the paddlewheels was evaluated at 18 kWh/ha-day[5]. Existing studies have produced a wide

range of values for paddlewheel energetic consumption [1, 4, 29, 30], due to variations in pond depth, mixing velocity, and process assumptions. Deviations in paddlewheel energetic consumption were included as a parameter in the sensitivity analysis.

5.3. PVC Liner

A 0.75 mm thick PVC membrane was assumed to line the ORPs with an average lifetime of 5 years[4]. The mass of PVC required to line the ORPs was calculated as the product of the surface area, thickness of the PVC membrane, and density of the PVC liner. The required surface area of the PVC liner was assumed to be 120% times the surface area of the cultivation ponds (500 ha)[3]. The density of the PVC membrane was taken to be 950 kg/m^3. The impacts of the PVC liner were normalized over the total amount of biomass produced over the lifetime of the PVC liner.

5.4. Freshwater Sourcing

The energy required to source freshwater to the ORPs was evaluated based off of conventional crop irrigation. It was assumed that electric pumps would bring surface and groundwater to the ORPs. The amount of energy required to source ground and surface water was based off of the 2008 Farm and ranch Irrigation survey[31], and the cost of electricity was assumed to be $0.10 kWh [3]

6. HARVESTING

6.1. Flocculation

Algae are pumped into post-cultivation holding tanks in which a coagulant, aluminum sulfate, is injected at a rate of 100 g/m^3 [32]. Aluminum sulfate was chosen for this study because it has been shown to be an effective coagulant for *Chlorella* algae [33]. Flocculation was assumed to concentrate the algal culture to a concentration of 2% (w/w). It was assumed that 90% of the medium from flocculation is recycled back to the cultivation ponds.

6.2. Pumping Requirements

Pumping power requirements (kWh/day) were constructed based on pipe flowrate (l/s), pipe diameter (m), pipe length (m), pipeline roughness (m), fluid velocity (m/s), pipe head-loss (m), Reynolds number (unit-less), and pump and motor efficiency (%).

The power requirement for pond pumping (J/s) is dependent on: g the gravitational acceleration (m/s^2), total lift (m), flow rate (m^3/s), density of fluid (kg/m^3), and motor efficiency (%) and is presented in Equation 17.

Equation 17

$$\boldsymbol{Power_{pump} = \frac{g*(Total\ Lift)*(Flowrate)*(density\ of\ fluid)}{Motor\ Efficiency}}$$

The Total Lift (m) is calculated as the sum of the Static Lift (m) and Pipe Head loss (m), shown in Equation 18.

Equation 18

$$\boldsymbol{Total\ Lift = Static\ Lift + Pipe\ Head\ loss}$$

Pipe head loss was based on the Darcy–Weisbach equation and is presented in Equation 19.

Equation 19

$$\boldsymbol{\frac{f*L*V^2}{2*g*D} = h_f}$$

Where h_f is head loss due to friction (m), L is the length of the pipe (m), V is the mean velocity of the flow (m/s), g is the acceleration due to gravity (m/s^2), D is the pipe diameter (m), and f is the Darcy-Weisbach friction factor.

The Swamee–Jain equation is used to solve for the Darcy-Weisbach friction factor *f,* and is presented in Equation 20 [34].

Equation 20

$$f = \frac{0.25}{[Log_{10}(\frac{\varepsilon}{3.7*D}+\frac{5.74}{Re^{0.9}})]^2}$$

Where ε is the pipeline roughness (m), Re is the Reynolds number for fluid flow in a pipe (unitless), and D is the diameter of the pipe (m)

The Reynolds number (Re) for fluid flow in a pipe is defined as:

Equation 21

$$Re = \frac{Q*D_H}{A*\nu}$$

Wherein Q is the volumetric flowrate (m^3/s), D_H is the hydraulic diameter of the pipe (m), A is the pipe cross sectional area (m^2), and ν is the kinematic viscosity (m^2/s).

The mean velocity of the flow (m/s) is expressed in Equation 22.

Equation 22

$$V = \frac{Q}{A}$$

Where A is the pipe cross sectional area (m^2), and Q is the volumetric flow rate (m^3/s).

The cross sectional area of a circular pipe is expressed in Equation 23

Equation 23

$$A = \pi * (\frac{D}{2})^2$$

Where D is the diameter of the pipe (m)

6.3. Centrifugation

After flocculation, algae are pumped to an industrial Centrifuge (CF) for dewatering. Decanter centrifuges were chosen as a means to concentrate the algae culture, as they are both a proven and reliable technology, and have the ability to significantly increase the concentration (% w/w) of the culture as compared to other centrifuge types. For centrifugation, the electrical consumption was evaluated at 8 kWh/m^3 consistent with centrifuges of this type[35]. Centrifugation was assumed to increase the algal concentration to 22% (w/w)[35]. In addition, it was assumed that 5% of the input culture would be lost during centrifugation, and that 90% of process medium would be recycled back into the ponds.

6.4. Chamber Filter Press

For Chamber Filter Presses (CFP), the electrical consumption per unit throughput was evaluated at 0.88 kWh/m^3 [35]. It was assumed that the chamber filter press would increase the algal concentration to 25% (w/w)[35]. It was assumed that 5% of the input culture would be lost during dewatering, and that 90% of the process medium would be recycled back into the ponds. The energetic and environmental costs associated with replacing the filter press membranes were not considered in this study.

6.5. Algal Drying

After dewatering, algae must undergo additional drying to achieve a final concentration of 90% (w/w). Two production scenarios were examined for drying: (i) natural gas based drying and (ii) waste heat drying

6.5.1. Natural Gas based Drying

(i): Algae from dewatering process are sent to an industrial boiler in which natural gas is burned to dry the algae. The amount of heat energy needed to dry the algae was based on the amount of water extracted from the system, latent heat of evaporation of water, and boiler efficiency. The boiler efficiency was assumed to be 75%, and it was assumed that 5% of the input algal biomass would be lost in this process.

The energy (kJ) required for Natural Gas based Drying (NGD) is dependent on: m the mass of water needed to be extracted from the system (kg), Cw the latent heat of evaporation of water (kJ/kg), Cv the specific heat of water (kJ/kg-C), ΔT the change in temperature of the water (C), and boiler efficiency ε_{NG} (%), expressed in Equation 24.

Equation 24 $$\boldsymbol{Energy_{heat} = m * (Cw + Cv * \Delta T)}$$

6.5.2. Waste Heat Drying

(ii): Studies have suggested that it may be possible to recover waste heat contained in the exhaust gases from power plants, and therefore utilize these exhaust streams to offset a portion of the energy required to dry the algal biomass[36]. Prior studies have estimated that a 500 MW power plant could generate up to $4.4*10^9$ MJ of "waste" heat energy per year[36], which greatly exceeds the heat energy required to dry the biomass. For Waste Heat Dying (WHD) scenarios it was assumed that all of the heat energy required to dry the biomass could be met using waste heat from a co-located power plant.

7. Life Cycle Inventory

The Life Cycle Inventory [LCI], normalized to 1 kg of biomass, for all production pathways are provided in the tables below. To avoid redundancy, the LCI of the following cultivation locations are provided: Mobile AL; Phoenix, AZ; San Diego, CA; Tallahassee, FL; Savannah, GA; Baton Rouge, LA; Brownsville, TX. In the following LCI tables these locations are referred to by state only. The sources of life cycle data are provided in the table below.

Table 18 – Sources of life cycle data

Input	Life Cycle Database
Aluminum Sulfate	Ecoinvent Database
PVC Liner	Ecoinvent Database
Wastewater	Ecoinvent Database
Urea	Ecoinvent Database
Superphosphate	Ecoinvent Database
Potassium Chloride	Ecoinvent Database
Electricity	USLCI Database
Natural Gas	USLCI Database

Table S19 – LCI normalized per kg biomass for MEA/CF/NGD production pathway for select locations

	MEA/Centrifugation/Natural Gas Based Drying													
	Freshwater Requirement	Wastewater Treatment	CO_2 Injected	PVC	Paddlewheels	Urea	SSP	KCL	Flocculation	MEA		Pumping and Sourcing Freshwater	CF	NGD
State	m^3 Freshwater	m^3 Process Water	kg CO_2	kg PVC	MJ Electricity	kg Urea	kg SSP	kg KCL	Kg $Al_2(SO_4)_3$	MJ Electricity	m3 Natural gas	MJ Electricity	MJ Electricity	m^3 Natural Gas
AL	0.42	0.36	3.00	0.03	0.37	0.26	0.10	0.04	0.25	0.35	0.50	1.34	1.56	0.38
AZ	0.72	0.33	3.00	0.02	0.28	0.26	0.10	0.04	0.25	0.35	0.50	2.42	1.56	0.38
CA	0.62	0.34	3.00	0.03	0.32	0.26	0.10	0.04	0.25	0.35	0.50	1.85	1.56	0.38
FL	0.39	0.35	3.00	0.03	0.35	0.26	0.10	0.04	0.25	0.35	0.50	1.61	1.56	0.38
GA	0.45	0.35	3.00	0.03	0.35	0.26	0.10	0.04	0.25	0.35	0.50	1.46	1.56	0.38
LA	0.43	0.36	3.00	0.03	0.36	0.26	0.10	0.04	0.25	0.35	0.50	1.77	1.56	0.38
TX	0.58	0.35	3.00	0.03	0.34	0.26	0.10	0.04	0.25	0.35	0.50	1.38	1.56	0.38

Table S20 – LCI normalized per kg biomass for MEA/CF/WHD production pathway for select locations

	MEA/Centrifugation/Waste Heat Drying													
	Freshwater Requirement	Wastewater Treatment	CO_2 Injected	PVC	Paddlewheels	Urea	SSP	KCL	Flocculation	MEA		Pumping and Sourcing Freshwater	CF	WHD
State	m^3 Freshwater	m^3 Process Water	kg CO_2	kg PVC	MJ Electricity	kg Urea	kg SSP	kg KCL	kg $Al_2(SO_4)_3$	MJ Electricity	m3 Natural gas	MJ Electricity	MJ Electricity	m^3 Natural Gas
AL	0.42	0.36	3.00	0.03	0.37	0.26	0.10	0.04	0.25	0.35	0.50	1.34	1.56	0
AZ	0.72	0.33	3.00	0.02	0.28	0.26	0.10	0.04	0.25	0.35	0.50	2.42	1.56	0
CA	0.62	0.34	3.00	0.03	0.32	0.26	0.10	0.04	0.25	0.35	0.50	1.85	1.56	0
FL	0.39	0.35	3.00	0.03	0.35	0.26	0.10	0.04	0.25	0.35	0.50	1.61	1.56	0
GA	0.45	0.35	3.00	0.03	0.35	0.26	0.10	0.04	0.25	0.35	0.50	1.46	1.56	0
LA	0.43	0.36	3.00	0.03	0.36	0.26	0.10	0.04	0.25	0.35	0.50	1.77	1.56	0
TX	0.58	0.35	3.00	0.03	0.34	0.26	0.10	0.04	0.25	0.35	0.50	1.38	1.56	0

Table S21 – LCI normalized per kg biomass for DI/CF/NGD production pathway for select locations

Direct Injection/Centrifugation/Natural Gas Based Drying													
	Freshwater Requirement	Wastewater Treatment	CO_2 Injected	PVC	Paddlewheels	Urea	SSP	KCL	Flocculation	DI	Pumping and Sourcing Freshwater	CF	NGD
**State**	m^3 Freshwater	m^3 Process Water	kg CO_2	kg PVC	MJ Electricity	kg Urea	kg SSP	kg KCL	kg $Al_2(SO_4)_3$	MJ Electricity	MJ Electricity	MJ Electricity	m^3 Natural Gas
**AL**	0.42	0.36	3.00	0.03	0.37	0.26	0.10	0.04	0.25	0.24	1.34	1.56	0.38
**AZ**	0.72	0.33	3.00	0.02	0.28	0.26	0.10	0.04	0.25	0.24	2.42	1.56	0.38
**CA**	0.62	0.34	3.00	0.03	0.32	0.26	0.10	0.04	0.25	0.24	1.85	1.56	0.38
**FL**	0.39	0.35	3.00	0.03	0.35	0.26	0.10	0.04	0.25	0.24	1.61	1.56	0.38
**GA**	0.45	0.35	3.00	0.03	0.35	0.26	0.10	0.04	0.25	0.24	1.46	1.56	0.38
**LA**	0.43	0.36	3.00	0.03	0.36	0.26	0.10	0.04	0.25	0.24	1.77	1.56	0.38
**TX**	0.58	0.35	3.00	0.03	0.34	0.26	0.10	0.04	0.25	0.24	1.38	1.56	0.38

Table S22 – LCI normalized per kg biomass for DI/CF/WHD production pathway for select locations

Direct Injection/Centrifugation/Waste Heat Drying													
	Freshwater Requirement	Wastewater Treatment	CO_2 Injected	PVC	Paddlewheels	Urea	SSP	KCL	Flocculation	DI	Pumping and Sourcing Freshwater	CF	WHD
**State**	m^3 Freshwater	m^3 Process Water	kg CO_2	kg PVC	MJ Electricity	kg Urea	kg SSP	kg KCL	kg $Al_2(SO_4)_3$	MJ Electricity	MJ Electricity	MJ Electricity	m^3 Natural Gas
**AL**	0.42	0.36	3.00	0.03	0.37	0.26	0.10	0.04	0.25	0.24	1.34	1.56	0
**AZ**	0.72	0.33	3.00	0.02	0.28	0.26	0.10	0.04	0.25	0.24	2.42	1.56	0
**CA**	0.62	0.34	3.00	0.03	0.32	0.26	0.10	0.04	0.25	0.24	1.85	1.56	0
**FL**	0.39	0.35	3.00	0.03	0.35	0.26	0.10	0.04	0.25	0.24	1.61	1.56	0
**GA**	0.45	0.35	3.00	0.03	0.35	0.26	0.10	0.04	0.25	0.24	1.46	1.56	0
**LA**	0.43	0.36	3.00	0.03	0.36	0.26	0.10	0.04	0.25	0.24	1.77	1.56	0
**TX**	0.58	0.35	3.00	0.03	0.34	0.26	0.10	0.04	0.25	0.24	1.38	1.56	0

Table S23 – LCI normalized per kg biomass for MEA/CFP/NGD production pathway for select locations

	MEA/Chamber Filter Press/Natural Gas Based Drying													
	Freshwater Requirement	Wastewater Treatment	CO_2 Injected	PVC	Paddlewheels	Urea	SSP	KCL	Flocculation	MEA		Pumping and Sourcing Freshwater	CFP	NGD
State	m^3 Freshwater	m^3 Process Water	kg CO_2	kg PVC	MJ Electricity	kg Urea	kg SSP	kg KCL	kg $Al_2(SO_4)_3$	MJ Electricity	m^3 natural gas	MJ Electricity	MJ Electricity	m^3 Natural Gas
AL	0.41	0.36	3.00	0.03	0.37	0.26	0.10	0.04	0.25	0.35	0.50	1.34	0.17	0.32
AZ	0.72	0.33	3.00	0.02	0.28	0.26	0.10	0.04	0.25	0.35	0.50	2.42	0.17	0.32
CA	0.61	0.34	3.00	0.03	0.32	0.26	0.10	0.04	0.25	0.35	0.50	1.85	0.17	0.32
FL	0.39	0.35	3.00	0.03	0.35	0.26	0.10	0.04	0.25	0.35	0.50	1.61	0.17	0.32
GA	0.45	0.35	3.00	0.03	0.35	0.26	0.10	0.04	0.25	0.35	0.50	1.46	0.17	0.32
LA	0.42	0.36	3.00	0.03	0.36	0.26	0.10	0.04	0.25	0.35	0.50	1.77	0.17	0.32
TX	0.57	0.35	3.00	0.03	0.34	0.26	0.10	0.04	0.25	0.35	0.50	1.38	0.17	0.32

Table S24 – LCI normalized per kg biomass for MEA/CFP/WHD production pathway for select locations

	MEA/Chamber Filter Press/Waste Heat Drying													
	Freshwater Requirement	Wastewater Treatment	CO_2 Injected	PVC	Paddlewheels	Urea	SSP	KCL	Flocculation	MEA		Pumping and Sourcing Freshwater	CFP	WHD
State	m^3 Freshwater	m^3 Process Water	kg CO_2	kg PVC	MJ Electricity	kg Urea	kg SSP	kg KCL	kg $Al_2(SO_4)_3$	MJ Electricity	m^3 natural gas	MJ Electricity	MJ Electricity	m^3 Natural Gas
AL	0.41	0.36	3.00	0.03	0.37	0.26	0.10	0.04	0.25	0.35	0.50	1.34	0.17	0
AZ	0.72	0.33	3.00	0.02	0.28	0.26	0.10	0.04	0.25	0.35	0.50	2.42	0.17	0
CA	0.61	0.34	3.00	0.03	0.32	0.26	0.10	0.04	0.25	0.35	0.50	1.85	0.17	0
FL	0.39	0.35	3.00	0.03	0.35	0.26	0.10	0.04	0.25	0.35	0.50	1.61	0.17	0
GA	0.45	0.35	3.00	0.03	0.35	0.26	0.10	0.04	0.25	0.35	0.50	1.46	0.17	0
LA	0.42	0.36	3.00	0.03	0.36	0.26	0.10	0.04	0.25	0.35	0.50	1.77	0.17	0
TX	0.57	0.35	3.00	0.03	0.34	0.26	0.10	0.04	0.25	0.35	0.50	1.38	0.17	0

Table S25 – LCI normalized per kg biomass for DI/CFP/NGD production pathway for select locations

Direct Injection/Chamber Filter Press/Natural Gas Based Drying													
	Freshwater Requirement	Wastewater Treatment	CO_2 Injected	PVC	Paddlewheels	Urea	SSP	KCL	Flocculation	DI	Pumping and Sourcing Freshwater	CFP	NGD
**State**	m^3 Freshwater	m^3 Process Water	kg CO_2	kg PVC	MJ Electricity	kg Urea	kg SSP	kg KCL	kg $Al_2(SO_4)_3$	MJ Electricity	MJ Electricity	MJ Electricity	m^3 Natural Gas
**AL**	0.41	0.36	3.00	0.03	0.37	0.26	0.10	0.04	0.25	0.24	1.34	0.17	0.32
**AZ**	0.72	0.33	3.00	0.02	0.28	0.26	0.10	0.04	0.25	0.24	2.42	0.17	0.32
**CA**	0.61	0.34	3.00	0.03	0.32	0.26	0.10	0.04	0.25	0.24	1.85	0.17	0.32
**FL**	0.39	0.35	3.00	0.03	0.35	0.26	0.10	0.04	0.25	0.24	1.61	0.17	0.32
**GA**	0.45	0.35	3.00	0.03	0.35	0.26	0.10	0.04	0.25	0.24	1.46	0.17	0.32
**LA**	0.42	0.36	3.00	0.03	0.36	0.26	0.10	0.04	0.25	0.24	1.77	0.17	0.32
**TX**	0.57	0.35	3.00	0.03	0.34	0.26	0.10	0.04	0.25	0.24	1.38	0.17	0.32

Table S26– LCI normalized per kg biomass for DI/CFP/WHD production pathway for select locations

Direct Injection/Chamber Filter Press/Waste Heat Drying													
	Freshwater Requirement	Wastewater Treatment	CO_2 Injected	PVC	Paddlewheels	Urea	SSP	KCL	Flocculation	DI	Pumping and Sourcing Freshwater	CFP	WHD
**State**	m^3 Freshwater	m^3 Process Water	kg CO_2	kg PVC	MJ Electricity	kg Urea	kg SSP	kg KCL	kg $Al_2(SO_4)_3$	MJ Electricity	MJ Electricity	MJ Electricity	m^3 Natural Gas
**AL**	0.41	0.36	3.00	0.03	0.37	0.26	0.10	0.04	0.25	0.24	1.34	0.17	0
**AZ**	0.72	0.33	3.00	0.02	0.28	0.26	0.10	0.04	0.25	0.24	2.42	0.17	0
**CA**	0.61	0.34	3.00	0.03	0.32	0.26	0.10	0.04	0.25	0.24	1.85	0.17	0
**FL**	0.39	0.35	3.00	0.03	0.35	0.26	0.10	0.04	0.25	0.24	1.61	0.17	0
**GA**	0.45	0.35	3.00	0.03	0.35	0.26	0.10	0.04	0.25	0.24	1.46	0.17	0
**LA**	0.42	0.36	3.00	0.03	0.36	0.26	0.10	0.04	0.25	0.24	1.77	0.17	0
**TX**	0.57	0.35	3.00	0.03	0.34	0.26	0.10	0.04	0.25	0.24	1.38	0.17	0

8. Direct Water Demands

The direct WDs for biomass production were calculated as the difference between the amount of make-up water required due to evaporation (W_{evap}), pond cleaning ($W_{cleaning}$), pond leaking ($W_{leaking}$), blowdown ($W_{blowdown}$), photosynthesis ($W_{photosyn}$), harvesting ($W_{harvest}$), algal drying (W_{drying}), water stored in the biomass that is transported offsite ($W_{biomass}$), and annual precipitation (W_{precip}), defined in Equation 25. A breakdown of the direct water demands for Chamber filter press based pathways for Phoenix, Arizona are provided in Figure 2.

Equation 25 $$Direct\ WD = W_{evap} + W_{cleaning} + W_{leaking} + W_{blowdown} + W_{photosyn} + W_{harvest} + W_{drying} + W_{biomass} - W_{precip}$$

Figure 2 – Direct Water Demands for CFP Pathways for Phoenix, Arizona

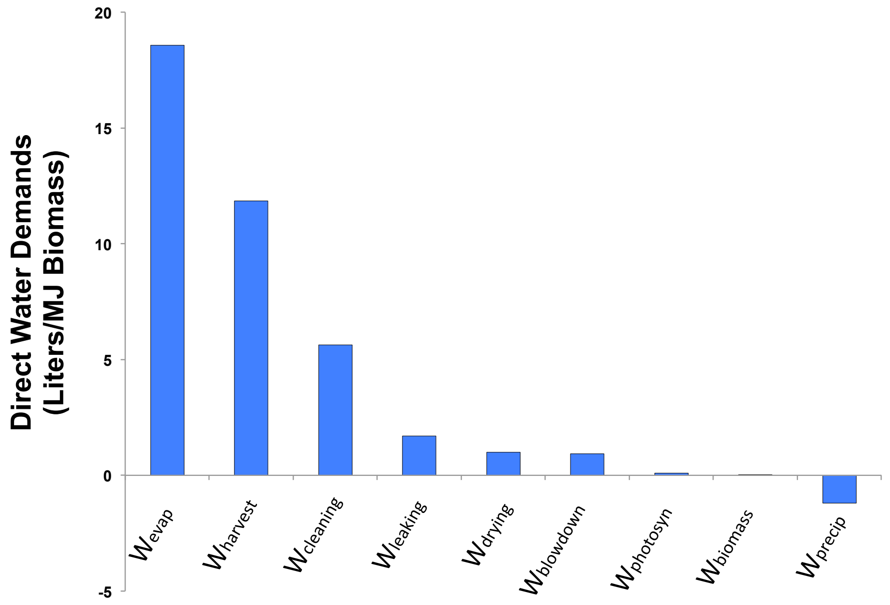

9. Allocation

In this study the energetic and environmental impacts of obtaining pure CO2 via MEA scrubbing are allocated between the algae cultivation facility and Natural Gas (NG) fired power plants on an energy basis. The allocation scheme is as follows: for every X MJ of electricity produced at a NG fired power plant, Y kg of CO2 are emitted which in turn produces Z MJ of algal biomass. The percentage of environmental and energetic impacts that are allocated to algal cultivation is equal to $\frac{Z}{(Z+X)}$. To estimate the amount of CO2 produced per MJ of electricity at a Natural Gas power plant, emissions data for over 450 NG fired power plants were acquired from the eGRID database. Analysis of this data indicates that on average NG fired power plant produce approximately 470 kg of CO2 per MWh of electricity. Under this assumption, 44.8% of the environmental impacts due to MEA are allocated to algae cultivation. Additionally, unallocated values for $EROI_{fossil}$ and life cycle GHG emissions are provided in summary tables in section 10.

10. Summary Tables

The following tables provide the direct WDs, $EROI_{fossil}$, and life cycle GHG emissions for biomass production with and without allocation.

Table S27 - Allocated $EROI_{fossil}$, net life cycle GHG emissions, and direct WDs for examined biomass production pathways & locations

Parameters		Cultivation and Harvesting Scenarios (Allocated)								WD[1]	
State	City	MEA/CF/NGD	MEA/CFP/NGD	DI/CF/ NGD	DI/CFP/NGD	MEA/CF/WHD	MEA/CFP/WHD	DI/CF/WHD	DI/CFP/WHD	CFP	CF
AL	Mobile	0.40 (44.2)	0.46 (22.5)	0.49 (18.9)	0.59 (-2.8)	0.60 (-0.4)	0.68 (-15.1)	0.86 (-25.7)	1.04 (-40.4)	22.1	22.3
AZ	Phoenix	0.38 (48.9)	0.43 (28.4)	0.47 (23.5)	0.56 (3.0)	0.57 (4.2)	0.64 (-9.2)	0.79 (-21.2)	0.94 (-34.6)	38.6	38.8
CA	San Diego	0.41 (32.0)	0.46 (16.0)	0.51 (6.3)	0.60 (-9.6)	0.63 (-12.6)	0.69 (-21.5)	0.91 (-38.3)	1.06 (-47.2)	32.8	33.0
FL	Daytona Beach	0.38 (43.0)	0.44 (22.7)	0.47 (17.5)	0.57 (-2.7)	0.58 (-1.6)	0.66 (-14.8)	0.81 (-27.1)	0.97 (-40.2)	24.1	24.3
FL	Jacksonville	0.38 (43.1)	0.44 (22.8)	0.47 (17.7)	0.57 (-2.6)	0.58 (-1.5)	0.66 (-14.7)	0.81 (-27.0)	0.97 (-40.1)	22.6	22.9
FL	Key West	0.38 (43.6)	0.44 (23.4)	0.47 (18.2)	0.57 (-2.1)	0.57 (-1.0)	0.65 (-14.2)	0.80 (-26.4)	0.97 (-39.6)	28.4	28.6
FL	Miami	0.38 (42.7)	0.44 (22.5)	0.48 (17.3)	0.57 (-3.0)	0.58 (-1.9)	0.66 (-15.1)	0.81 (-27.3)	0.98 (-40.5)	22.1	22.4
FL	Tallahassee	0.39 (42.4)	0.44 (22.2)	0.48 (17.0)	0.57 (-3.2)	0.58 (-2.2)	0.66 (-15.4)	0.82 (-27.6)	0.98 (-40.8)	20.8	21.1
FL	Tampa	0.38 (43.2)	0.44 (23.0)	0.47 (17.8)	0.57 (-2.5)	0.58 (-1.4)	0.65 (-14.6)	0.81 (-26.8)	0.97 (-40.0)	25.1	25.4
FL	West Palm Beach	0.38 (43.0)	0.44 (22.7)	0.47 (17.6)	0.57 (-2.7)	0.58 (-1.6)	0.66 (-14.8)	0.81 (-27.0)	0.97 (-40.2)	22.9	23.1
GA	Savannah	0.39 (45.0)	0.45 (23.2)	0.49 (19.7)	0.59 (-2.1)	0.60 (0.4)	0.68 (-14.3)	0.86 (-24.9)	1.03 (-39.6)	24.1	24.4
LA	Baton Rouge	0.39 (39.2)	0.45 (20.8)	0.49 (13.6)	0.58 (-4.7)	0.60 (-5.4)	0.67 (-16.8)	0.86 (-31.0)	1.01 (-42.3)	22.6	22.8
LA	Lake Charles	0.39 (39.0)	0.45 (20.6)	0.49 (13.5)	0.58 (-4.9)	0.60 (-5.6)	0.67 (-16.9)	0.86 (-31.1)	1.02 (-42.4)	23.0	23.2
LA	New Orleans	0.40 (38.9)	0.45 (20.5)	0.49 (13.4)	0.58 (-5.0)	0.60 (-5.7)	0.67 (-17.0)	0.86 (-31.2)	1.02 (-42.5)	22.1	22.3
TX	Austin	0.39 (41.5)	0.45 (20.6)	0.49 (16.1)	0.59 (-4.8)	0.59 (-3.1)	0.68 (-16.9)	0.85 (-28.5)	1.03 (-42.3)	29.8	30.0
TX	Brownsville	0.39 (41.8)	0.45 (20.9)	0.49 (16.4)	0.58 (-4.5)	0.59 (-2.8)	0.68 (-16.6)	0.84 (-28.2)	1.02 (-42.0)	30.6	30.8
TX	Corpus Christi	0.39 (42.1)	0.45 (21.3)	0.48 (16.8)	0.58 (-4.1)	0.59 (-2.5)	0.67 (-16.3)	0.84 (-27.8)	1.02 (-41.7)	29.7	29.9
TX	Houston	0.39 (42.0)	0.45 (21.1)	0.49 (16.7)	0.58 (-4.2)	0.59 (-2.6)	0.67 (-16.4)	0.84 (-27.9)	1.02 (-41.8)	25.6	25.8
TX	Lufkin	0.39 (41.5)	0.45 (20.6)	0.49 (16.1)	0.59 (-4.8)	0.59 (-3.1)	0.68 (-16.9)	0.85 (-28.5)	1.03 (-42.3)	26.1	26.3
TX	Port Arthur	0.40 (35.2)	0.46 (16.9)	0.51 (9.7)	0.60 (-8.6)	0.62 (-9.4)	0.70 (-20.6)	0.91 (-34.9)	1.08 (-46.2)	22.8	23.0
TX	San Antonio	0.39 (41.3)	0.45 (20.4)	0.49 (16.0)	0.59 (-4.9)	0.60 (-3.3)	0.68 (-17.1)	0.85 (-28.6)	1.03 (-42.5)	29.9	30.1
TX	Victoria	0.39 (41.9)	0.45 (21.0)	0.49 (16.5)	0.58 (-4.4)	0.59 (-2.7)	0.68 (-16.5)	0.84 (-28.1)	1.02 (-41.9)	27.4	27.7

Table S28 - Unallocated EROI$_{fossil}$, net life cycle GHG emissions, and direct WDs for examined biomass production pathways & locations

Parameters		Cultivation and Harvesting Scenarios (Unallocated)								WD[1]	
State	City	MEA/CF/NGD	MEA/CFP/NGD	MEA/CF/WHD	MEA/CFP/WHD	DI/CF/ NGD	DI/CFP/NGD	DI/CF/WHD	DI/CFP/WHD	CFP	CF
AL	Mobile	0.31 (78.6)	0.35 (56.8)	0.43 (33.9)	0.47 (19.2)	0.49 (18.9)	0.59 (-2.8)	0.86 (-25.7)	1.04 (-40.4)	22.1	22.3
AZ	Phoenix	0.30 (83.0)	0.34 (62.5)	0.41 (38.4)	0.45 (25.0)	0.47 (23.5)	0.56 (3.0)	0.79 (-21.2)	0.94 (-34.6)	38.6	38.8
CA	San Diego	0.32 (65.5)	0.35 (49.5)	0.44 (20.9)	0.48 (12.0)	0.51 (6.3)	0.60 (-9.6)	0.91 (-38.3)	1.06 (-47.2)	32.8	33.0
FL	Daytona Beach	0.31 (77.1)	0.34 (56.8)	0.42 (32.4)	0.46 (19.3)	0.47 (17.5)	0.57 (-2.7)	0.81 (-27.1)	0.97 (-40.2)	24.1	24.3
FL	Jacksonville	0.31 (77.2)	0.34 (56.9)	0.42 (32.6)	0.46 (19.4)	0.47 (17.7)	0.57 (-2.6)	0.81 (-27.0)	0.97 (-40.1)	22.6	22.9
FL	Key West	0.31 (77.7)	0.34 (57.5)	0.42 (33.1)	0.46 (19.9)	0.47 (18.2)	0.57 (-2.1)	0.80 (-26.4)	0.97 (-39.6)	28.4	28.6
FL	Miami	0.31 (76.8)	0.34 (56.6)	0.42 (32.2)	0.46 (19.0)	0.48 (17.3)	0.57 (-3.0)	0.81 (-27.3)	0.98 (-40.5)	22.1	22.4
FL	Tallahassee	0.31 (76.5)	0.34 (56.3)	0.42 (31.9)	0.46 (18.7)	0.48 (17.0)	0.57 (-3.2)	0.82 (-27.6)	0.98 (-40.8)	20.8	21.1
FL	Tampa	0.31 (77.3)	0.34 (57.1)	0.42 (32.7)	0.46 (19.5)	0.47 (17.8)	0.57 (-2.5)	0.81 (-26.8)	0.97 (-40.0)	25.1	25.4
FL	West Palm Beach	0.31 (77.1)	0.34 (56.8)	0.42 (32.5)	0.46 (19.3)	0.47 (17.6)	0.57 (-2.7)	0.81 (-27.0)	0.97 (-40.2)	22.9	23.1
GA	Savannah	0.31 (79.3)	0.35 (57.5)	0.43 (34.7)	0.47 (20.0)	0.49 (19.7)	0.59 (-2.1)	0.86 (-24.9)	1.03 (-39.6)	24.1	24.4
LA	Baton Rouge	0.31 (73.0)	0.35 (54.6)	0.43 (28.4)	0.47 (17.1)	0.49 (13.6)	0.58 (-4.7)	0.86 (-31.0)	1.01 (-42.3)	22.6	22.8
LA	Lake Charles	0.31 (72.8)	0.35 (54.5)	0.43 (28.2)	0.47 (16.9)	0.49 (13.5)	0.58 (-4.9)	0.86 (-31.1)	1.02 (-42.4)	23.0	23.2
LA	New Orleans	0.31 (72.7)	0.35 (54.4)	0.43 (28.1)	0.47 (16.8)	0.49 (13.4)	0.58 (-5.0)	0.86 (-31.2)	1.02 (-42.5)	22.1	22.3
TX	Austin	0.31 (75.7)	0.35 (54.8)	0.43 (31.1)	0.47 (17.3)	0.49 (16.1)	0.59 (-4.8)	0.85 (-28.5)	1.03 (-42.3)	29.8	30.0
TX	Brownsville	0.31 (76.0)	0.35 (55.1)	0.43 (31.4)	0.47 (17.6)	0.49 (16.4)	0.58 (-4.5)	0.84 (-28.2)	1.02 (-42.0)	30.6	30.8
TX	Corpus Christi	0.31 (76.3)	0.35 (55.5)	0.43 (31.7)	0.47 (17.9)	0.48 (16.8)	0.58 (-4.1)	0.84 (-27.8)	1.02 (-41.7)	29.7	29.9
TX	Houston	0.31 (76.2)	0.35 (55.3)	0.43 (31.6)	0.47 (17.8)	0.49 (16.7)	0.58 (-4.2)	0.84 (-27.9)	1.02 (-41.8)	25.6	25.8
TX	Lufkin	0.31 (75.7)	0.35 (54.8)	0.43 (31.1)	0.47 (17.3)	0.49 (16.1)	0.59 (-4.8)	0.85 (-28.5)	1.03 (-42.3)	26.1	26.3
TX	Port Arthur	0.32 (69.1)	0.36 (50.7)	0.44 (24.5)	0.48 (13.2)	0.51 (9.7)	0.60 (-8.6)	0.91 (-34.9)	1.08 (-46.2)	22.8	23.0
TX	San Antonio	0.31 (75.7)	0.35 (54.6)	0.43 (30.9)	0.47 (17.1)	0.49 (16.0)	0.59 (-4.9)	0.85 (-28.6)	1.03 (-42.5)	29.9	30.1
TX	Victoria	0.31 (76.1)	0.35 (55.2)	0.43 (31.5)	0.47 (17.6)	0.49 (16.5)	0.58 (-4.4)	0.84 (-28.1)	1.02 (-41.9)	27.4	27.7

* The results are presented in the following format: EROI$_{fossil}$ (Net Life Cycle GHG Emissions) where Net life cycle GHG emissions are in units of (g CO_2 eq/MJ-Biomass)

[1] The results for the WD are presented in units of (liters/MJ-biomass)

11. Additional Scenarios

11.1. Low Nitrogen Scenario

Previous studies have suggested that cultivating algae in low-nitrogen conditions can substantially increase the lipid content of the biomass[37-39]. However, nitrogen deprivation may have adverse effects on the algae culture, and additional experimental data is required to validate the feasibility of this approach. A low-nitrogen scenario was considered in this analysis, the fractionalized composition of algae under nitrogen deprivation, taken from Lardon et al. 2009, was evaluated at 38.5% lipids, 52.9% carbohydrates, and 6.7% proteins[40]. The following tables provide the $EROI_{fossil}$, and life cycle GHG emissions for biomass production for the low-nitrogen scenario (with and without allocation).

Table S29 – Low Nitrogen Scenario: Allocated $EROI_{fossil}$ & net life cycle GHG emissions for examined biomass production pathways & locations

Parameters		Cultivation and Harvesting Scenarios (Allocated)							
**State**	**City**	**MEA/CF/NGD**	**MEA/CFP/NGD**	**DI/CF/ NGD**	**DI/CFP/NGD**	**MEA/CF/WHD**	**MEA/CFP/WHD**	**DI/CF/WHD**	**DI/CFP/WHD**
**AL**	Mobile	0.54 (22.8)	0.63 (4.8)	0.72 (-1.6)	0.90 (-19.5)	0.87 (-14.0)	1.02 (-26.1)	1.50 (-38.3)	1.97 (-50.4)
**AZ**	Phoenix	0.51 (27.3)	0.59 (10.5)	0.68 (3.0)	0.83 (-13.9)	0.81 (-9.4)	0.92 (-20.4)	1.31 (-33.8)	1.65 (-44.8)
**CA**	San Diego	0.55 (12.4)	0.64 (-0.7)	0.75 (-12.1)	0.92 (-25.2)	0.91 (-24.3)	1.03 (-31.6)	1.62 (-48.8)	2.04 (-56.1)
**FL**	Daytona Beach	0.52 (21.8)	0.61 (5.1)	0.69 (-2.6)	0.86 (-19.3)	0.83 (-15.0)	0.96 (-25.8)	1.36 (-39.3)	1.78 (-50.2)
**FL**	Jacksonville	0.52 (21.9)	0.61 (5.2)	0.69 (-2.5)	0.86 (-19.2)	0.82 (-14.9)	0.96 (-25.7)	1.36 (-39.2)	1.77 (-50.1)
**FL**	Key West	0.52 (22.4)	0.60 (5.7)	0.68 (-2.0)	0.85 (-18.6)	0.82 (-14.3)	0.95 (-25.2)	1.34 (-38.7)	1.75 (-49.6)
**FL**	Miami	0.52 (21.5)	0.61 (4.8)	0.69 (-2.9)	0.86 (-19.5)	0.83 (-15.2)	0.97 (-26.1)	1.37 (-39.6)	1.79 (-50.4)
**FL**	Tallahassee	0.52 (21.2)	0.61 (4.6)	0.69 (-3.1)	0.86 (-19.8)	0.83 (-15.5)	0.97 (-26.4)	1.38 (-39.9)	1.81 (-50.7)
**FL**	Tampa	0.52 (22.0)	0.61 (5.3)	0.69 (-2.4)	0.86 (-19.0)	0.82 (-14.7)	0.96 (-25.6)	1.35 (-39.1)	1.77 (-50.0)
**FL**	West Palm Beach	0.52 (21.8)	0.61 (5.1)	0.69 (-2.6)	0.86 (-19.3)	0.82 (-14.9)	0.96 (-25.8)	1.36 (-39.3)	1.78 (-50.2)
**GA**	Savannah	0.53 (23.4)	0.63 (5.5)	0.72 (-0.9)	0.89 (-18.8)	0.87 (-13.3)	1.01 (-25.4)	1.48 (-37.6)	1.94 (-49.7)
**LA**	Baton Rouge	0.53 (18.6)	0.62 (3.5)	0.72 (-5.8)	0.88 (-21.0)	0.87 (-18.1)	0.99 (-27.5)	1.48 (-42.6)	1.89 (-51.9)
**LA**	Lake Charles	0.54 (18.4)	0.62 (3.3)	0.72 (-6.0)	0.88 (-21.1)	0.87 (-18.3)	0.99 (-27.6)	1.49 (-42.7)	1.90 (-52.1)
**LA**	New Orleans	0.54 (18.3)	0.62 (3.2)	0.72 (-6.1)	0.89 (-21.3)	0.87 (-18.4)	1.00 (-27.7)	1.49 (-42.8)	1.90 (-52.2)
**TX**	Austin	0.53 (20.4)	0.63 (3.2)	0.71 (-4.0)	0.89 (-21.2)	0.86 (-16.3)	1.01 (-27.7)	1.46 (-40.7)	1.94 (-52.1)
**TX**	Brownsville	0.53 (20.7)	0.62 (3.5)	0.71 (-3.7)	0.89 (-20.9)	0.86 (-16.1)	1.00 (-27.4)	1.45 (-40.4)	1.92 (-51.8)
**TX**	Corpus Christi	0.53 (21.0)	0.62 (3.8)	0.71 (-3.3)	0.89 (-20.5)	0.85 (-15.7)	1.00 (-27.1)	1.44 (-40.1)	1.90 (-51.4)
**TX**	Houston	0.53 (20.9)	0.62 (3.7)	0.71 (-3.4)	0.89 (-20.6)	0.85 (-15.8)	1.00 (-27.2)	1.44 (-40.2)	1.91 (-51.6)
**TX**	Lufkin	0.53 (20.4)	0.63 (3.2)	0.71 (-4.0)	0.89 (-21.2)	0.86 (-16.4)	1.01 (-27.8)	1.46 (-40.7)	1.94 (-52.1)
**TX**	Port Arthur	0.55 (15.0)	0.64 (-0.1)	0.75 (-9.4)	0.93 (-24.5)	0.91 (-21.7)	1.05 (-31.0)	1.62 (-46.1)	2.11 (-55.4)
**TX**	San Antonio	0.53 (20.2)	0.63 (3.0)	0.71 (-4.1)	0.90 (-21.3)	0.86 (-16.5)	1.01 (-27.9)	1.46 (-40.9)	1.95 (-52.3)
**TX**	Victoria	0.53 (20.7)	0.62 (3.5)	0.71 (-3.6)	0.89 (-20.8)	0.85 (-16.0)	1.00 (-27.4)	1.44 (-40.3)	1.92 (-51.7)

Table S30 - Low Nitrogen Scenario: Unllocated EROI$_{fossil}$ & net life cycle GHG emissions for examined biomass production pathways & locations

Parameters		Cultivation and Harvesting Scenarios (Unallocated)							
**State**	**City**	**MEA/CF/NGD**	**MEA/CFP/NGD**	**MEA/CF/WHD**	**MEA/CFP/WHD**	**DI/CF/ NGD**	**DI/CFP/NGD**	**DI/CF/WHD**	**DI/CFP/WHD**
**AL**	Mobile	0.42 (49.6)	0.48 (31.7)	0.60 (12.9)	0.67 (0.8)	0.72 (-1.6)	0.90 (-19.5)	1.50 (-38.3)	1.97 (-50.4)
**AZ**	Phoenix	0.40 (54.0)	0.45 (37.2)	0.57 (17.3)	0.63 (6.3)	0.68 (2.9)	0.83 (-13.9)	1.31 (-33.8)	1.65 (-44.8)
**CA**	San Diego	0.43 (38.6)	0.48 (25.5)	0.62 (1.9)	0.68 (-5.4)	0.75 (-12.1)	0.92 (-25.2)	1.62 (-48.8)	2.04 (-56.1)
**FL**	Daytona Beach	0.41 (48.4)	0.46 (31.8)	0.58 (11.7)	0.64 (0.9)	0.69 (-2.7)	0.86 (-19.3)	1.36 (-39.3)	1.78 (-50.2)
**FL**	Jacksonville	0.41 (48.5)	0.46 (31.9)	0.58 (11.8)	0.64 (1.0)	0.69 (-2.5)	0.86 (-19.2)	1.36 (-39.2)	1.77 (-50.1)
**FL**	Key West	0.41 (49.1)	0.46 (32.4)	0.58 (12.4)	0.64 (1.5)	0.68 (-2.0)	0.85 (-18.6)	1.34 (-38.7)	1.75 (-49.6)
**FL**	Miami	0.41 (48.2)	0.46 (31.5)	0.58 (11.5)	0.64 (0.6)	0.69 (-2.9)	0.86 (-19.5)	1.37 (-39.6)	1.79 (-50.4)
**FL**	Tallahassee	0.41 (47.9)	0.46 (31.2)	0.58 (11.2)	0.65 (0.3)	0.69 (-3.2)	0.86 (-19.8)	1.38 (-39.9)	1.81 (-50.7)
**FL**	Tampa	0.41 (48.7)	0.46 (32.0)	0.58 (12.0)	0.64 (1.1)	0.69 (-2.4)	0.86 (-19.0)	1.35 (-39.1)	1.77 (-50.0)
**FL**	West Palm Beach	0.41 (48.4)	0.46 (31.8)	0.58 (11.7)	0.64 (0.9)	0.69 (-2.6)	0.86 (-19.3)	1.36 (-39.3)	1.78 (-50.2)
**GA**	Savannah	0.42 (50.2)	0.47 (32.3)	0.60 (13.5)	0.66 (1.4)	0.72 (-0.9)	0.89 (-18.8)	1.48 (-37.6)	1.94(-49.7)
**LA**	Baton Rouge	0.42 (45.0)	0.47 (29.9)	0.60 (8.4)	0.66 (-1.0)	0.72 (-5.9)	0.88 (-21.0)	1.48 (-42.6)	1.89 (-51.9)
**LA**	Lake Charles	0.42 (44.9)	0.47 (29.8)	0.60 (8.2)	0.66 (-1.1)	0.72 (-6.0)	0.88 (-21.1)	1.49 (-42.7)	1.90 (-52.1)
**LA**	New Orleans	0.42 (44.8)	0.47 (29.7)	0.60 (8.1)	0.66 (-1.2)	0.72 (-6.1)	0.89 (-21.3)	1.49 (-42.8)	1.90 (-52.2)
**TX**	Austin	0.42 (47.1)	0.47 (29.9)	0.60 (10.4)	0.66 (-1.0)	0.71 (-4.0)	0.89 (-21.2)	1.46 (-40.7)	1.94 (-52.1)
**TX**	Brownsville	0.42 (47.4)	0.47 (30.2)	0.59 (10.7)	0.66 (-0.7)	0.71 (-3.7)	0.89 (-20.9)	1.45 (-40.4)	1.92 (-51.8)
**TX**	Corpus Christi	0.42 (47.7)	0.47 (30.6)	0.59 (11.1)	0.66 (-0.3)	0.71 (-3.4)	0.89 (-20.5)	1.44 (-40.1)	1.90 (-51.4)
**TX**	Houston	0.42 (47.6)	0.47 (30.5)	0.59 (10.9)	0.66 (-0.4)	0.71 (-3.5)	0.89 (-20.6)	1.44 (-40.2)	1.91 (-51.6)
**TX**	Lufkin	0.42 (47.1)	0.47 (29.9)	0.60 (10.4)	0.66 (-1.0)	0.71 (-4.0)	0.89 (-21.2)	1.46 (-40.7)	1.94 (-52.1)
**TX**	Port Arthur	0.43 (41.5)	0.48 (26.4)	0.62 (4.8)	0.68 (-4.5)	0.75 (-9.5)	0.93 (-24.5)	1.62 (-46.1)	2.11 (-55.4)
**TX**	San Antonio	0.42 (46.9)	0.47 (29.8)	0.60 (10.2)	0.66 (-1.1)	0.71 (-4.2)	0.90 (-21.3)	1.46 (-40.9)	1.95 (-52.3)
**TX**	Victoria	0.42 (47.5)	0.47 (30.3)	0.59 (10.8)	0.66 (-0.6)	0.71 (-3.6)	0.89 (-20.8)	1.44 (-40.3)	1.92 (-51.7)

* The results are presented in the following format: EROI$_{fossil}$ (Net Life Cycle GHG Emissions) where Net life cycle GHG emissions are in units of (g CO_2 eq/MJ-Biomass)

11.2. Alternative Production Scenario

An alternate technological route was considered in this work. This production pathway assumes that algae undergo auto-flocculation (AF) to concentrate the biomass to .25% (w/w) [41]. Recent studies have suggested that cross flow filtration (CFF) is a low-energy intensive technology that can be used to dewater the algae culture, and has many advantages over conventional centrifugation, pressure filtration, and dissolved air and/or froth flotation[42]. Therefore, post auto-flocculation biomass is then sent to a cross-flow filtration unit for further dewatering to 16% (w/w); the electrical consumption for cross flow filtration was evaluated at .5 kWh/m^3 [42]. A chamber filter press (CFP) is then used to further concentrate the algae to 25% (w/w), and both natural gas (NG) and waste heat (WHD) are evaluated as processing options for drying the biomass to 90% (w/w). This technological route may be favorable, as it does not rely on a coagulant for biomass production and uses low-energy dewatering strategies. The results are presented in Table S31 and Table S32.

Table S31 – Alternate Biomass Production Scenario: Allocated $EROI_{fossil}$ & net life cycle GHG emissions for examined biomass production pathways & locations

Parameters		Cultivation and Harvesting Scenarios (Allocated)							
Growth Conditions		Normal Growth Conditions				Low Nitrogen Scenario			
**State**	**City**	**MEA/AF/CFF/ CFP/NG**	**DI/AF/CFF/ CFP/NG**	**MEA/AF/CFF/ CFP/WHD**	**DI/AF/CFF/ CFP/ WHD**	**MEA/AF/CFF/ CFP/NG**	**DI/AF/CFF/ CFP/NG**	**MEA/AF/CFF/ CFP/WHD**	**DI/AF/CFF/ CFP/ WHD**
**AL**	Mobile	0.45 (14.6)	0.59 (-10.7)	0.68 (-22.9)	1.04 (-48.2)	0.63 (-1.6)	0.90 (-25.9)	1.01 (-32.5)	1.96 (-56.8)
**AZ**	Phoenix	0.43 (20.7)	0.56 (-4.7)	0.64 (-16.8)	0.94 (-42.2)	0.59 (4.2)	0.83 (-20.2)	0.92 (-26.7)	1.65 (-51.1)
**CA**	San Diego	0.46 (9.0)	0.60 (-16.7)	0.70 (-28.6)	1.08 (-54.2)	0.64 (-6.5)	0.93 (-31.1)	1.05 (-37.4)	2.10 (-61.9)
**FL**	Daytona Beach	0.44 (15.1)	0.57 (-10.3)	0.65 (-22.5)	0.97 (-47.9)	0.61 (-1.2)	0.85 (-25.6)	0.95 (-32.1)	1.76 (-56.5)
**FL**	Jacksonville	0.44 (15.2)	0.57 (-10.2)	0.65 (-22.3)	0.96 (-47.8)	0.61 (-1.1)	0.85 (-25.5)	0.95 (-32.0)	1.75 (-56.4)
**FL**	Key West	0.44 (15.7)	0.56 (-9.7)	0.65 (-21.8)	0.96 (-47.2)	0.60 (-0.6)	0.85 (-24.9)	0.95 (-31.5)	1.72 (-55.8)
**FL**	Miami	0.44 (14.8)	0.57 (-10.6)	0.65 (-22.7)	0.97 (-48.1)	0.61 (-1.5)	0.86 (-25.8)	0.96 (-32.3)	1.77 (-56.7)
**FL**	Tallahassee	0.44 (14.5)	0.57 (-10.9)	0.66 (-23.0)	0.97 (-48.4)	0.61 (-1.7)	0.86 (-26.1)	0.96 (-32.6)	1.78 (-57.0)
**FL**	Tampa	0.44 (15.3)	0.56 (-10.1)	0.65 (-22.2)	0.96 (-47.6)	0.60 (-1.0)	0.85 (-25.3)	0.95 (-31.9)	1.74 (-56.2)
**FL**	West Palm Beach	0.44 (15.1)	0.57 (-10.3)	0.65 (-22.4)	0.97 (-47.8)	0.61 (-1.2)	0.85 (-25.6)	0.95 (-32.1)	1.75 (-56.5)
**GA**	Savannah	0.45 (15.4)	0.59 (-9.9)	0.68 (-22.1)	1.03 (-47.5)	0.63 (-1.0)	0.89 (-25.3)	1.00 (-31.9)	1.93 (-56.2)
**LA**	Baton Rouge	0.45 (13.4)	0.58 (-12.1)	0.67 (-24.1)	1.02 (-49.6)	0.62 (-2.6)	0.89 (-27.1)	1.00 (-33.5)	1.91 (-58.0)
**LA**	Lake Charles	0.45 (13.2)	0.58 (-12.3)	0.68 (-24.3)	1.02 (-49.8)	0.62 (-2.8)	0.89 (-27.2)	1.00 (-33.7)	1.92 (-58.1)
**LA**	New Orleans	0.45 (13.1)	0.58 (-12.4)	0.68 (-24.4)	1.02 (-49.9)	0.62 (-2.9)	0.89 (-27.3)	1.00 (-33.8)	1.92 (-58.2)
**TX**	Austin	0.45 (12.9)	0.58 (-12.5)	0.67 (-24.6)	1.02 (-50.0)	0.62 (-3.2)	0.89 (-27.5)	1.00 (-34.1)	1.91 (-58.4)
**TX**	Brownsville	0.45 (13.2)	0.58(-12.2)	0.67 (-24.3)	1.01 (-49.7)	0.62 (-2.9)	0.88 (-27.2)	0.99 (-33.8)	1.89 (-58.1)
**TX**	Corpus Christi	0.45 (13.5)	0.58 (-11.9)	0.67 (-24.0)	1.01 (-49.4)	0.62 (-2.6)	0.88 (-26.9)	0.99 (-33.4)	1.88 (-57.8)
**TX**	Houston	0.45 (13.4)	0.58 (-12.0)	0.67 (-24.1)	1.01 (-49.5)	0.62 (-2.7)	0.88 (-27.0)	0.99 (-33.6)	1.88 (-57.9)
**TX**	Lufkin	0.45 (12.9)	0.58 (-12.5)	0.67 (-24.6)	1.02 (-50.0)	0.62 (-3.2)	0.89 (-27.6)	1.00 (-34.1)	1.91 (-58.4)
**TX**	Port Arthur	0.46 (9.5)	0.60 (-16.0)	0.70 (-28.0)	1.09 (-53.5)	0.64 (-6.2)	0.93 (-30.6)	1.05 (-37.0)	2.13 (-61.5)
**TX**	San Antonio	0.45 (12.7)	0.58 (-12.7)	0.68 (-24.8)	1.02 (-50.2)	0.62 (-3.4)	0.89 (-27.7)	1.00 (-34.3)	1.92 (-58.6)
**TX**	Victoria	0.45 (13.3)	0.58 (-12.1)	0.67 (-24.3)	1.01 (-49.6)	0.62 (-2.8)	0.88 (-27.2)	0.99 (-33.7)	1.89 (-58.1)

*The results are presented in the following format: $EROI_{fossil}$ (Net Life Cycle GHG Emissions) where Net life cycle GHG emissions are in units of (g CO_2 eq/MJ Biomass)

Table S32 – Alternate Biomass Production Scenario: Allocated $EROI_{fossil}$ & net life cycle GHG emissions for examined biomass production pathways & locations

**Parameters**		**Cultivation and Harvesting Scenarios (Unallocated)**							
**Growth Conditions**		**Normal Growth Conditions**				**Low Nitrogen Scenario**			
**State**	**City**	**MEA/AF/CFF/CFP/NG**	**MEA/AF/CFF/CFP/WHD**	**DI/AF/CFF/CFP/NG**	**DI/AF/CFF/CFP/ WHD**	**MEA/AF/CFF/CFP/NG**	**MEA/AF/CFF/CFP/WHD**	**DI/AF/CFF/CFP/NG**	**DI/AF/CFF/CFP/ WHD**
**AL**	Mobile	0.35 (48.9)	0.47 (11.4)	0.59 (-10.7)	1.04 (-48.2)	0.48 (25.2)	0.67 (-5.7)	0.90 (-25.9)	1.96 (-56.8)
**AZ**	Phoenix	0.34 (54.9)	0.45 (17.3)	0.56 (-4.7)	0.94 (-42.2)	0.45 (30.9)	0.63 (0.0)	0.83 (-20.2)	1.65 (-51.1)
**CA**	San Diego	0.36 (42.5)	0.48 (4.9)	0.60 (-16.7)	1.08 (-54.2)	0.48 (19.7)	0.68 (-11.2)	0.93 (-31.1)	2.10 (-61.9)
**FL**	Daytona Beach	0.34 (49.2)	0.46 (11.6)	0.57 (-10.3)	0.97 (-47.9)	0.46 (25.5)	0.64 (-5.4)	0.85 (-25.6)	1.76 (-56.5)
**FL**	Jacksonville	0.34 (49.3)	0.46 (11.7)	0.57 (-10.2)	0.96 (-47.8)	0.46 (25.6)	0.64 (-5.3)	0.85 (-25.5)	1.75 (-56.4)
**FL**	Key West	0.34 (49.8)	0.45 (12.3)	0.56 (-9.7)	0.96 (-47.2)	0.46 (26.1)	0.64 (-4.8)	0.85 (-24.9)	1.72 (-55.8)
**FL**	Miami	0.34 (48.9)	0.46 (11.4)	0.57 (-10.6)	0.97 (-48.1)	0.46 (25.2)	0.64 (-5.7)	0.86 (-25.8)	1.77 (-56.7)
**FL**	Tallahassee	0.34 (48.6)	0.46 (11.1)	0.57 (-10.9)	0.97 (-48.4)	0.46 (25.0)	0.64 (-5.9)	0.86 (-26.1)	1.78 (-57.0)
**FL**	Tampa	0.34 (49.4)	0.46 (11.9)	0.56 (-10.1)	0.96 (-47.6)	0.46 (25.7)	0.64 (-5.2)	0.85 (-25.3)	1.74 (-56.2)
**FL**	West Palm Beach	0.34 (49.2)	0.46 (11.7)	0.57 (-10.3)	0.97 (-47.8)	0.46 (25.5)	0.64 (-5.4)	0.85 (-25.6)	1.75 (-56.5)
**GA**	Savannah	0.35 (49.7)	0.47 (12.2)	0.59 (-9.9)	1.03 (-47.5)	0.47 (25.9)	0.66 (-5.0)	0.89 (-25.3)	1.93 (-56.2)
**LA**	Baton Rouge	0.35 (47.2)	0.47 (9.7)	0.58 (-12.1)	1.02 (-49.6)	0.47 (23.9)	0.66 (-7.0)	0.89 (-27.1)	1.91 (-58.0)
**LA**	Lake Charles	0.35 (47.1)	0.47 (9.5)	0.58 (-12.3)	1.02 (-49.8)	0.47 (23.7)	0.66 (-7.2)	0.89 (-27.2)	1.92 (-58.1)
**LA**	New Orleans	0.35 (47.0)	0.47 (9.4)	0.58 (-12.4)	1.02 (-49.9)	0.47 (23.6)	0.66 (-7.3)	0.89 (-27.3)	1.92 (-58.2)
**TX**	Austin	0.35 (47.1)	0.47 (9.6)	0.58 (-12.5)	1.02 (-50.0)	0.47 (23.6)	0.66 (-7.3)	0.89 (-27.5)	1.91 (-58.4)
**TX**	Brownsville	0.35 (47.4)	0.47 (9.9)	0.58(-12.2)	1.01 (-49.7)	0.47 (23.9)	0.66 (-7.0)	0.88 (-27.2)	1.89 (-58.1)
**TX**	Corpus Christi	0.35 (47.7)	0.46 (10.2)	0.58 (-11.9)	1.01 (-49.4)	0.47 (24.2)	0.65 (-6.7)	0.88 (-26.9)	1.88 (-57.8)
**TX**	Houston	0.35 (47.6)	0.47 (10.1)	0.58 (-12.0)	1.01 (-49.5)	0.47 (24.1)	0.66 (-6.8)	0.88 (-27.0)	1.88 (-57.9)
**TX**	Lufkin	0.35 (47.1)	0.47 (9.5)	0.58 (-12.5)	1.02 (-50.0)	0.47 (23.6)	0.66 (-7.3)	0.89 (-27.6)	1.91 (-58.4)
**TX**	Port Arthur	0.36 (43.4)	0.48 (5.8)	0.60 (-16.0)	1.09 (-53.5)	0.48 (20.3)	0.68 (-10.6)	0.93 (-30.6)	2.13 (-61.5)
**TX**	San Antonio	0.35 (46.9)	0.47 (9.4)	0.58 (-12.7)	1.02 (-50.2)	0.47 (23.4)	0.66 (-7.5)	0.89 (-27.7)	1.92 (-58.6)
**TX**	Victoria	0.35 (47.4)	0.47 (9.9)	0.58 (-12.1)	1.01 (-49.6)	0.47 (23.9)	0.66 (-6.9)	0.88 (-27.2)	1.89 (-58.1)

* The results are presented in the following format: $EROI_{fossil}$ (Net Life Cycle GHG Emissions) where Net life cycle GHG emissions are in units of (g CO_2 eq/MJ-Biomass)

12. Reference $EROI_{fossil}$

$EROI_{fossil}$ for biofuels derived from various feedstock's are provided in the table below. As seen from Table S33, the $EROI_{fossil}$ for algal biofuels can range from 0.29-2.49 depending on whether wet or dry extraction is implemented. The results presented in this work assume that algal biomass will have to be dried to 90% (w/w) before further downstream process of the biomass to biofuel is possible, and are comparable to the results found for producing biomass using the nominal dry route presented in ref[43].

Table S33 – Reference $EROI_{fossil}$ for various biofuels

Feedstock	Fuel Product	$EROI_{fossil}$	Source	Notes:
Rapeseed oil	Biodiesel	2.29	[44]	Study applicable to UK
Rapeseed oil	Biodiesel	3.0	[44]	Study applicable to Europe
Jatropha oil	Biodiesel	1.9	[44]	Study applicable to India
Palm oil	Biodiesel	7.8-10.3	[44]	Study applicable to Brazil
Palm oil	Biodiesel	5.9-6.9	[44]	Study applicable to Colombia
Recycled frying oil	Biodiesel	5.51	[44]	Study applicable to Germany
Waste vegetable oil	Biodiesel	7.8	[44]	Study applicable to USA
Waste vegetable oil	Biodiesel	7.96	[44]	Study applicable to Spain
Soybean	Biodiesel	5.54	[7]	Year: 2011
Algae	Biodiesel	1.87	[4]	Assumes wet extraction
Algae	Biodiesel	1.82	[45]	Assumes wet extraction
Algae	Hydrocarbon biofuels	2.49	[43]	Nominal wet route
Algae	Hydrocarbon biofuels	0.29	[43]	Nominal dry route
Algae	Biomass	0.86	[43]	Nominal dry route
Algae	Biomass	0.38 - 1.08	This study	Normal Culture Conditions[1]
Algae	Biomass	0.43 – 2.13	This study	Low N Scenario[1]

[1] Allocation is performed

13. Citations

1. Clarens AF, Resurreccion EP, White MA, Colosi LM: **Environmental life cycle comparison of algae to other bioenergy feedstocks.** *Environ Sci Technol* 2010, **44:**1813-1819.
2. Uduman N, Qi Y, Danquah MK, Forde GM, Hoadley A: **Dewatering of microalgal cultures: A major bottleneck to algae-based fuels.** *J Renew Sustain Energy* 2010, **2**.
3. Murphy CF, Allen DT: **Energy-water nexus for mass cultivation of algae.** *Environ Sci Technol* 2011, **45:**5861-5868.
4. Stephenson AL, Kazamia E, Dennis JS, Howe CJ, Scott SA, Smith AG: **Life-Cycle Assessment of Potential Algal Biodiesel Production in the United Kingdom: A Comparison of Raceways and Air-Lift Tubular Bioreactors.** *Energy & Fuels* 2010, **24:**4062-4077.
5. Oswald BJ: **Systems and Economic Analysis of Microalgae Ponds for Conversion of CO2 to Biomass - Final Report.** In *Book Systems and Economic Analysis of Microalgae Ponds for Conversion of CO2 to Biomass - Final Report* (Editor ed.^eds.). City; 1996.
6. Xu L, Weathers PJ, Xiong XR, Liu CZ: **Microalgal bioreactors: Challenges and opportunities.** *Eng Life Sci* 2009, **9:**178-189.
7. Pradhan A, Shrestha, D.S., Mcaloon, A.J., Yee, W.C., Haas, M.J., Duffield, J.A. : **Energy life-cycle assessment of soybean biodiesel revisited.** *American Society of Agricultural and Biological Engineers* 2011, **54:**1031-1039.
8. Sheehan J, Laboratory NRE: *Life Cycle Inventory of Biodiesel and Petroleum Diesel for Use in an Urban Bus: Final Report, May 1998.* National Renewable Energy Laboratory; 1998.
9. International Organization for Standardization: **ISO 14040 Environmental management - Life cycle assessment - Principles and framework.** In *Book ISO 14040 Environmental management - Life cycle assessment - Principles and framework* (Editor ed.^eds.). City; 2006.
10. **Power Profiler** [http://www.epa.gov/cleanenergy/energy-and-you/how-clean.html]
11. NREL: **National Solar Radiation Database (1961-1990).** In *Book National Solar Radiation Database (1961-1990)* (Editor ed.^eds.). City; 1994.
12. Weyer KM, Bush DR, Darzins A, Willson BD: **Theoretical Maximum Algal Oil Production.** *Bioenergy Research* 2010, **3:**204-213.
13. Shuttleworth WJ: **Putting the "vap" into evaporation.** *Hydrol EarthSyst Sci* 2007.
14. Merva GE: *Physio-engineering Principles.* Westport,CT: AVI Publishing Company; 1975.
15. NOAA: **Monthly Average Precipitation by State.** In *Book Monthly Average Precipitation by State* (Editor ed.^eds.). City: National Climatic Data Center, National Oceanic and Atmospheric Administration; 2006.
16. Lardon L, Hélias A, Sialve B, Steyer J-P, Bernard O: **Life-cycle assessment of biodiesel production from microalgae.** *Environ Sci Technol* 2009, **43:**6475-6481.
17. Brune DE, Lundquist TJ, Benemann JR: *Microalgal Biomass for Greenhouse Gas Reductions: Potential for Replacement of Fossil Fuels and Animal Feeds.* ASCE; 2009.
18. E.W B: **Micro-algae as a source of protein.** *Biotechnology Advances*, **25:**207-210.
19. Becker EW: *Microalgae: biotechnology and microbiology.* Cambridge University Press; 1993.

20. Mata TM, Martins AA, Caetano NS: **Microalgae for biodiesel production and other applications: a review.** *Renewable and Sustainable Energy Reviews* 2010, **14:**217-232.
21. Cogley JG: **The Albedo of Water as a Function of Latitude.** *Monthly Weather Review* 1979, **107:**775-781.
22. Goldman JC: **OUTDOOR ALGAL MASS-CULTURES .2. PHOTOSYNTHETIC YIELD LIMITATIONS.** *Water Res* 1979, **13:**119-136.
23. Krassen D: **GreenFuel Technologies: A Case Study for Industrial Photosynthetic Energy Capture.** In *Book GreenFuel Technologies: A Case Study for Industrial Photosynthetic Energy Capture* (Editor ed.^eds.). City.
24. Kadam KL: **Environmental implications of power generation via coal-microalgae cofiring.** *Energy* 2002, **27:**905-922.
25. Benemann JR: **CO_2 mitigation with microalgae systems.** *Energy Conv Manag* 1997, **38:**S475-S479.
26. Ho SH, Chen CY, Lee DJ, Chang JS: **Perspectives on microalgal CO(2)-emission mitigation systems - A review.** *Biotechnology Advances* 2011, **29:**189-198.
27. Cho S, Lee D, Luong TT, Park S, Oh YK, Lee T: **Effects of Carbon and Nitrogen Sources on Fatty Acid Contents and Composition in the Green Microalga, Chlorella sp 227.** *J Microbiol Biotechnol* 2011, **21:**1073-1080.
28. Lv JM, Cheng LH, Xu XH, Zhang L, Chen HL: **Enhanced lipid production of Chlorella vulgaris by adjustment of cultivation conditions.** *Bioresour Technol* 2010, **101:**6797-6804.
29. Kadam KL: **Microalgae production from power plant flue gas: Environmental implications on life cycle basis.** In *Book Microalgae production from power plant flue gas: Environmental implications on life cycle basis* (Editor ed.^eds.). City: National Renewable Energy Laboratory; 2001.
30. Collet P, Hélias A, Lardon L, Ras M, Goy R-A, Steyer J-P: **Life-cycle assessment of microalgae culture coupled to biogas production.** *Bioresour Technol* 2011, **102:**207-214.
31. Farm and Rach Irrigation Survey (2008): **2007 Census of Agriculture.** In *Book 2007 Census of Agriculture* (Editor ed.^eds.). City.
32. Shelef G, Sukenik A, Green M: **Microalgae Harvesting and Processing: A Literature Review.** In *Book Microalgae Harvesting and Processing: A Literature Review* (Editor ed.^eds.). City: Solar Energy Research Institute; 1984.
33. Golueke CG OW: **Harvesting and processing sewage gorwn plaktonic algae.** 1965.
34. Swamee Pk, Jain, A.K.: **Explicit equations for pipe-flow problems.** *Journal of the Hydraulics Division* 1976.
35. FH M: *Experiences and Strategies in the recovery of biomass from mass cultures of microalgae.* Amsterdam: Elsevier; 1980.
36. Clarens AF, Nassau H, Resurreccion EP, White MA, Colosi LM: **Environmental impacts of algae-derived biodiesel and bioelectricity for transportation.** *Environ Sci Technol* 2011, **45:**7554-7560.
37. Converti A, Casazza AA, Ortiz EY, Perego P, Del Borghi M: **Effect of temperature and nitrogen concentration on the growth and lipid content of Nannochloropsis oculata and Chlorella vulgaris for biodiesel production.** *Chemical Engineering and Processing: Process Intensification* 2009, **48:**1146-1151.

38. Illman AM, Scragg AH, Shales SW: **Increase in Chlorella strains calorific values when grown in low nitrogen medium.** *Enzyme and Microbial Technology* 2000, **27:**631-635.
39. Flynn KJ, Garrido JL, Zapata M, Öpik H, Hipkin CR: **Changes in fatty acids, amino acids and carbon/nitrogen biomass during nitrogen starvation of ammonium- and nitrate-grownIsochrysis galbana.** *J Appl Phycol* 1992, **4:**95-104.
40. Lardon L, Helias A, Sialve B, Steyer J-P, Bernard O: **Life-Cycle Assessment of Biodiesel Production from Microalgae.** *Environmental Science & Technology* 2009**:**7.
41. Sukenik A, Shelef G: **Algal autoflocculation—verification and proposed mechanism.** *Biotechnology and bioengineering* 1984, **26:**142-147.
42. Bhave R, Kuritz T, Powell L, Adcock D: **Membrane-Based Energy Efficient Dewatering of Microalgae in Biofuels Production and Recovery of Value Added Co-Products.** *Environ Sci Technol* 2012, **46:**5599-5606.
43. Vasudevan V, Stratton RW, Pearlson MN, Jersey GR, Beyene AG, Weissman JC, Rubino M, Hileman JI: **Environmental Performance of Algal Biofuel Technology Options.** *Environ Sci Technol* 2012, **46:**2451-2459.
44. Khanal SK, Environmental, Bioenergy WRI, Committee BT: *Bioenergy and Biofuel from Biowastes and Biomass.* Amer Society of Civil Engineers; 2010.
45. Frank ED, Han J, Palou-Rivera I, Elgowainy A, Wang MQ: **Methane and nitrous oxide emissions affect the life-cycle analysis of algal biofuels.** *Environmental Research Letters* 2012, **7:**014030.
